# Connecting the Dots: How MicroRNAs Link Asthma and Atherosclerosis

**DOI:** 10.3390/ijms26083570

**Published:** 2025-04-10

**Authors:** Răzvan-Ionuț Zimbru, Elena-Larisa Zimbru, Florina-Maria Bojin, Laura Haidar, Minodora Andor, Octavia Oana Harich, Gabriela Tănasie, Carmen Tatu, Diana-Evelyne Mailat, Iulia-Maria Zbîrcea, Bogdan Hirtie, Cristina Uța, Camelia-Felicia Bănărescu, Carmen Panaitescu

**Affiliations:** 1 Center of Immuno-Physiology and Biotechnologies, Department of Functional Sciences, “Victor Babes” University of Medicine and Pharmacy, 300041 Timisoara, Romania; razvan.zimbru@umft.ro (R.-I.Z.);; 2 Research Center for Gene and Cellular Therapies in the Treatment of Cancer—OncoGen, Timis County Emergency Clinical Hospital “Pius Brinzeu”, 156 Liviu Rebreanu Bd., 300723 Timisoara, Romania; 3 Multidisciplinary Heart Research Center, “Victor Babes” University of Medicine and Pharmacy, 300041 Timisoara, Romania; 4 Timis County Emergency Clinical Hospital “Pius Brinzeu”, 156 Liviu Rebreanu Bd., 300723 Timisoara, Romania; 5 Cardiology Clinic, Timisoara Municipal Clinical Emergency Hospital, 12 Revoluției din 1989 Bd., 300040 Timisoara, Romania; 6 Department of Automation and Applied Informatics, “Politehnica” University of Timisoara, 300006 Timișoara, Romania; 7 ENT Department, “Victor Babes” University of Medicine and Pharmacy, 300042 Timișoara, Romania

**Keywords:** microRNA (miRNA), inflammation, asthma, atherosclerosis, agomir and antagomir therapy

## Abstract

Asthma and atherosclerosis are chronic conditions with distinct pathophysiologies, but overlapping inflammatory mechanisms that suggest a potential common regulatory framework. MicroRNAs (miRNAs), small non-coding RNA molecules that modulate gene expression post-transcriptionally, could be key players in linking these disorders. This review outlines how miRNAs contribute to the complex interplay between asthma and atherosclerosis, focusing on key miRNAs involved in inflammatory pathways, immune cell regulation and vascular remodeling. We discuss specific miRNAs, such as miR-155, miR-21 and miR-146a, which have been shown to modulate inflammatory cytokine production and T cell differentiation, impacting respiratory and cardiovascular health. The common miRNAs found in both asthma and atherosclerosis emphasize their role as potential biomarkers, but also as therapeutic targets. Understanding these molecular connections may unlock novel approaches for innovative, integrated treatment strategies that address both conditions and may significantly improve patient outcomes. Further research is needed to explore mechanistic pathways and validate the translational potential of miRNA-based interventions in preclinical and clinical settings.

## 1. Introduction

Cardiovascular disease (CVD) continues to be the leading cause of mortality globally, and recent studies project a significant increase in CVD prevalence and associated risk factors in the coming decades [1]. Atherosclerosis is recognized as a major contributor to the development of CVD, with ischemic heart disease and stroke being the leading causes of the global CVD burden [2,3]. Inflammation is widely acknowledged as a fundamental factor in the initiation and progression of atherosclerosis, influencing each stage from early endothelial dysfunction to the formation of advanced plaques [4,5]. This complex disease is characterized by the formation of arterial lesions caused by subendothelial lipid accumulation and chronic low-grade inflammation in the middle and large arteries, mostly driven by macrophages various subsets of T cells [6,7,8]. Among the T cells involved, T helper 1 (Th1) cells play a pivotal role by secreting pro-inflammatory cytokines such as interferon-gamma (IFN-γ), which exacerbate endothelial dysfunction and promote macrophage activation. Regulatory T cells (Tregs), on the other hand, have a protective role by suppressing inflammatory responses and maintaining immune tolerance, thereby regulating plaque progression. Additionally, T helper 17 (Th17) cells contribute to the inflammatory milieu through the production of interleukin (IL)-17, which further promotes vascular inflammation and plaque instability. The interplay between these T cell subsets and macrophages drives the chronic inflammatory environment that sustains the development and progression of atherosclerotic plaques [9]. Specifically, modified Low-Density Lipoprotein (LDL) is taken up by monocyte-derived macrophages, forming foam cells and attracting additional inflammatory cells to the arterial intima. Inflammatory mechanisms are involved in all stages of atherosclerotic lesion development, linking dyslipidemia to the formation of vulnerable plaques that can result in complications such as ischemic heart disease or stroke. Both innate and adaptive immune responses play crucial roles in plaque development [7,8].

Atherosclerosis is significantly influenced by vascular smooth muscle cells (vSMCs), which undergo phenotypic switching—a pathological transformation that allows vSMCs to dedifferentiate, migrate and adopt proliferative, pro-inflammatory and pro-migratory features [10,11].

Treatment for atherosclerosis focuses on lifestyle modifications, blood pressure management, statins and antiplatelet agents. Statins are the primary lipid-lowering therapy, and also provide pleiotropic benefits, such as anti-inflammatory and plaque stabilization effects. However, a residual cardiovascular risk persists in many patients despite optimal statin doses, like in those with diabetes, genetic predisposition and chronic inflammation [12,13]. To address these challenges, new approaches have been explored, including proprotein convertase subtilisin/kexin type 9 (PCSK9) inhibitors, which enhance LDL receptor recycling to lower LDL-C levels. Other targets include apolipoprotein CIII (APOC3), lipoprotein(a) (Lp(a)) and angiopoietin-like protein 3 (ANGPTL3), all of which have effects on lipid dysregulation and atherogenesis [14,15,16]. Anti-inflammatory therapies targeting cytokines such as tumor necrosis factor-α (TNF-α), IL-1β and the nucleotide-binding domain, leucine-rich family, pyrin domain-containing 3 (NLRP3) inflammasome have shown promise. For instance, the CANTOS (Canakinumab Anti-Inflammatory Thrombosis Outcome Study) trial demonstrated improved cardiovascular outcomes with the anti-IL-1β antibody canakinumab, independent of lipid levels. Similarly, colchicine, an anti-inflammatory drug that targets the NLRP3 inflammasome/IL-1β-IL-6 axis, has been effective in reducing cardiac events in patients with atherosclerosis in trials like COLCOT (Colchicine Cardiovascular Outcomes Trial) and LoDoCo2 (Low-Dose Colchicine 2 Trial) [17,18,19].

Non-coding RNAs (ncRNAs), including microRNAs (miRNAs), interfere with key processes such as regulation of endothelial cell function and vascular smooth muscle cells, cholesterol homeostasis, immune cell recruitment and modulation of inflammation [10,20,21]. These small, non-coding RNA molecules regulate gene expression at the post-transcriptional level by binding to target messenger RNA (mRNA). Understanding the complex role of miRNAs and their interactions could reveal new therapeutic targets for the treatment and prevention of atherosclerotic cardiovascular diseases [22,23,24].

Asthma is a chronic inflammatory condition of the airways, characterized by symptoms such as shortness of breath, chest tightness, wheezing and coughing, that arises from a complex interplay of environmental and genetic factors [9,25,26,27].

Asthma is a heterogeneous disease characterized by varying underlying biological mechanisms, known as endotypes, which drive its development and progression [28]. The most well-defined category is type 2 (T2) inflammation, which includes allergic and eosinophilic asthma. This endotype is marked by elevated levels of type 2 cytokines (IL-4, IL-5, IL-13), alarmins (IL-25, IL-33, TSLP) and key biomarkers such as plasma eosinophils, immunoglobulin E (IgE) and fractional exhaled nitric oxide (FeNO) [9].

In contrast, non-type 2 (T2-low) inflammation encompasses endotypes driven by alternative pathways, such as neutrophilic inflammation, metabolic dysfunction or paucigranulocytic profiles [29,30]. For example, obesity-related asthma is linked to systemic inflammation and metabolic changes [31].

Understanding these endotypes is essential for advancing precision medicine, as it enables the development of targeted therapies, such as biologic therapies that inhibit specific cytokines (e.g., anti-IL-5, anti-IgE) for T2-high asthma, while highlighting the need for novel treatments for T2-low subtypes. Further elucidating the complex interplay of immune and molecular mechanisms will not only refine existing therapies, but also lead to innovative approaches to improve patient care [32,33,34].

MiRNA involvement in essential processes in asthma, such as smooth muscle cell proliferation, mucus production, collagen deposition and airway hyperactivity, emphasize their potential as therapeutic targets. Unique miRNA profiles have been identified within different T helper cell subpopulations, such as Th1, Th2, Th17 and Treg cells. These miRNA signatures not only indicate the activation status of these cells, but also correlate with the inflammatory characteristics of various asthma phenotypes and their severity [9,23,35]. MiRNAs, small non-coding RNAs that regulate gene expression, have emerged as promising tools in this context. MiRNAs associated with T2 asthma, such as miR-21, miR-145 and miR-155, are involved in modulating immune responses and airway inflammation. These miRNAs can improve diagnostic accuracy, monitor disease progression and guide personalized treatments [23,36].

In contrast, non-type 2 (T2-low) asthma encompasses phenotypes primarily driven by Th1 and Th17 immune responses. One prominent phenotype is neutrophilic asthma, characterized by increased neutrophils in the blood and sputum. MiRNAs, such as miR-223 and miR-146a, have been implicated in neutrophilic inflammation, and may provide insights into the underlying mechanisms of T2-low asthma. Further research into miRNAs and their regulatory networks could contribute to establishing clear guidelines for their use as diagnostic biomarkers, while also enabling the development of novel targeted therapies to improve patient outcomes in these complex phenotypes [9,27].

Understanding the molecular mechanisms underlying severe asthma is essential for better disease management and treatment options [23,36,37,38]. Importantly, individuals with chronic inflammatory conditions such as asthma may also be at an increased risk of developing CVD, due to the persistent systemic inflammatory state and shared molecular pathways [39,40].

**Figure 1:**
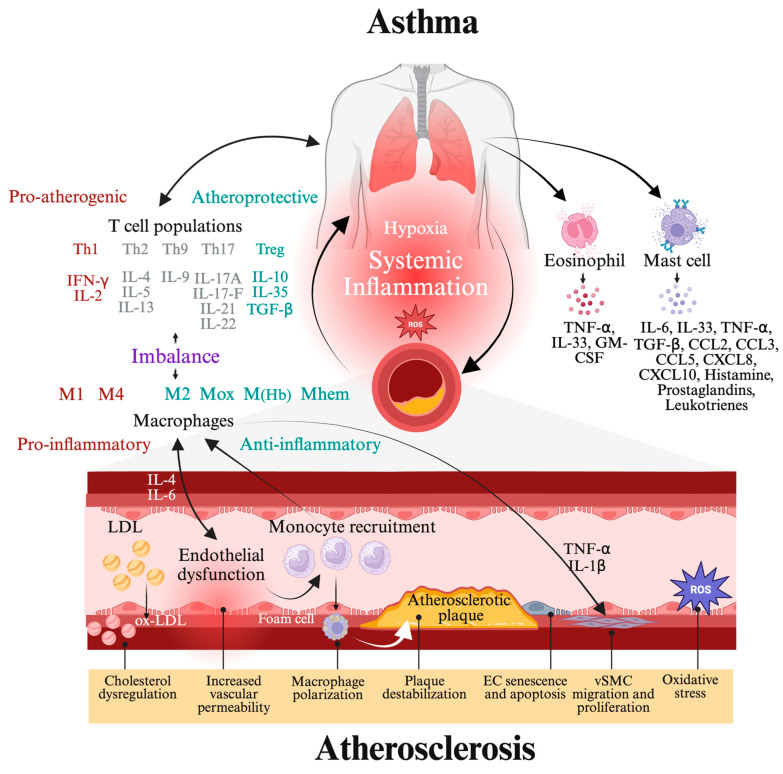
Potential pathogenetic mechanisms of connecting chronic airway inflammation in asthma to atherosclerotic cardiovascular disease. This figure illustrates the interconnected systemic inflammatory mechanisms between asthma and atherosclerosis. Chronic airway inflammation in asthma is driven by activated immune cells such as eosinophils, mast cells and various types of T cell populations, each producing a range of pro-inflammatory cytokines (e.g., IL-4, IL-5, IL-6, IL-13, IL-17, TNF-α, IL-33, GM-CSF), therefore illustrating the immune imbalance that drives inflammatory signaling. The resulting systemic inflammation and hypoxia contribute to endothelial dysfunction and promote vascular inflammation. This imbalance affects macrophage polarization, promoting inflammatory subtypes (M1, M4) over anti-inflammatory phenotypes (M2, Mox, M(Hb), Mhem), further driving vascular inflammation and plaque formation. These immune alterations, along with elevated oxidative stress (ROS) and cytokine signaling (e.g., IL-1β, TNF-α), contribute to LDL oxidation, foam cell formation and, ultimately, to atherosclerotic plaque formation and progression. The bottom panel highlights the downstream vascular consequences, including endothelial senescence and apoptosis, vascular smooth muscle cell alterations and increased plaque instability, which, together, may accelerate the development of cardiovascular disease (CVD) in asthma patients. TNF-α: tumor necrosis factor-α, IL: interleukin, GM-CSF: Granulocyte-Macrophage Colony-Stimulating Factor, TGF-β: Transforming Growth Factor-β, CCL: C-C Motif Chemokine Ligand, CXCL: C-X-C Motif Chemokine Ligand, LDL: Low-Density Lipoprotein, ox-LDL: Oxidized Low-Density Lipoprotein, EC: endothelial cell, vSMC: vascular smooth muscle cell, ROS: reactive oxygen species. Created with BioRender.com.

Asthma and atherosclerosis are two seemingly unrelated chronic diseases, yet both affect millions of people worldwide [41]. Despite affecting different organ systems, emerging studies reveal that they share overlapping inflammatory mechanisms, highlighting potential connections between these seemingly distinct conditions (Figure 1) [42,43,44]. However, recent research suggests that they are linked through common pathophysiological processes, including inflammatory pathways, immune dysregulation, molecular signaling pathways and even genetic predispositions [41,45,46,47]. Chronic inflammation plays a central role in both conditions, with immune cells such as macrophages, T cells and mast cells contributing to disease progression. Additionally, systemic inflammation in asthma, driven by cytokines like IL-6, IL-13 and TNF-α, has been linked to endothelial dysfunction and vascular damage, key processes in atherosclerosis [41]. Epidemiological studies further support this link, showing that individuals with asthma have a higher risk of developing cardiovascular diseases, including atherosclerosis [46,48]. The presence of chronic inflammation and oxidative stress in asthma can accelerate atherosclerotic plaque formation, while endothelial dysfunction in atherosclerosis may exacerbate airway hyper-responsiveness [47].

This narrative review explores the shared inflammatory mechanisms underlying asthma and atherosclerosis, with a particular focus on the regulatory role of miRNAs. Specifically, we examine key miRNAs involved in inflammatory pathways, immune cell regulation and vascular remodeling, emphasizing their potential as biomarkers and therapeutic targets. Additionally, this review aims to describe both shared and distinct miRNA pathways in these conditions, and evaluate the therapeutic potential of agomir/antagomir-based strategies. A comprehensive literature search was conducted using PubMed, Scopus and Web of Science, employing predefined keywords related to asthma, atherosclerosis and microRNAs. Studies were selected based on their relevance to inflammatory mechanisms and their publication date. To ensure rigor, references were thoroughly cross-checked for consistency and alignment with our research objectives.

## 2. MicroRNAs at the Crossroads of Human Health and Disease

**Figure 2:**
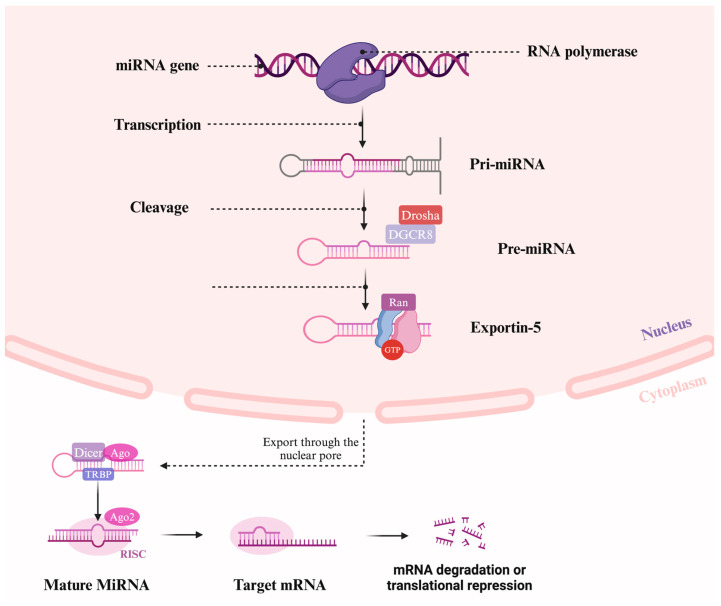
A schematic representation of the biogenesis and function of microRNAs. The process begins with the transcription of miRNA genes into primary miRNAs (pri-miRNAs), which are then cleaved into precursor miRNAs (pre-miRNAs). These pre-miRNAs are exported from the nucleus to the cytoplasm by Exportin-5. In the cytoplasm, pre-miRNAs undergo further processing to become mature miRNAs. The mature miRNAs are incorporated into the RNA-induced silencing complex (RISC), where they guide the complex to target mRNAs, leading to mRNA degradation or translational repression, thereby regulating gene expression. Created with BioRender.com.

MiRNAs are a prominent class of highly conserved, single-stranded non-coding RNAs, approximately between 18 and 25 nucleotides in length, that regulate gene expression at the post-transcriptional level by either inhibiting the translation of messenger RNA (mRNA) into protein or promoting the degradation of mRNA (Figure 2) [20,49].

MiRNAs exert their effects through base pairing with specific sequences in the 3′ untranslated region (3′ UTR) of target mRNAs, thereby influencing the transcriptome and proteome of eukaryotic cells. Currently, around 2600 microRNAs have been identified in humans [50,51,52]. Many of these non-coding RNAs are located within the introns of protein-coding genes, and are thought to regulate the expression of about 33% of cellular genes involved in processes such as differentiation, proliferation, migration, senescence, apoptosis and angiogenesis. As critical regulators, miRNAs play essential roles in a wide array of biological functions, including cell growth, development and programmed cell death [51,52]. Disruptions in miRNA expression and function are closely associated with a range of human diseases, including cancer, neurological and neurodegenerative disorders, pulmonary conditions such as asthma and chronic obstructive pulmonary disease (COPD), and metabolic disorders like diabetes mellitus and obesity, as well as cardiovascular diseases such as atherosclerosis, hypertension and heart failure [22,35,53,54]. Their intricate effects in individuals with multiple comorbid conditions represent a particularly compelling area for further research, offering potentially new perspectives into common mechanisms and opportunities for integrated therapeutic strategies. The biogenesis of miRNAs begins with the processing of transcripts generated by RNA polymerase II or III, which occurs either during or after transcription. A significant proportion of all known miRNAs are intragenic, derived mainly from intronic regions and, to a lesser extent, from exonic sequences of protein-coding genes. On the other hand, intergenic miRNAs are transcribed independently of a host gene, under the control of their own promoters. In some cases, multiple miRNAs are transcribed as a single precursor transcript, forming clusters that often share seed regions, thereby grouping them into the same family. The biogenesis of miRNAs follows either a canonical or necanonical processing pathway [24].

The biogenesis and function of miRNAs have been largely explained within the past two decades, revealing the canonical pathway that involves key proteins, such as DiGeorge syndrome chromosomal region 8 (DGCR8), ribonuclease III (RNase III) enzyme (DROSHA) and cytoplasmic ribonuclease III (RNase III) enzyme (Dicer) (Figure 2) [53]. Most miRNA genes are transcribed by RNA Polymerase II, producing long primary miRNAs (pri-miRNAs) ranging from 500 to 3000 base pairs, which are processed into pre-miRNAs (miRNAs precursor form) in the nucleus [55,56]. Pre-miRNAs are transported to the cytoplasm by exportin 5 (Exp5), where Dicer cleaves them into mature miRNA duplexes [57]. Of the two strands produced, the one with lower stability at the 5′ end, known as the guide strand, is preferentially selected and incorporated into the RNA-induced silencing complex (RISC). This process is facilitated by proteins such as transactivation-responsive RNA-binding protein (TRBP) and PACT, an activator of interferon-induced protein kinase R (PKR) [58]. The guide strand within the RISC silences gene expression by repressing mRNA translation or inducing mRNA degradation via cleavage or deadenylation. The other strand, termed the passenger strand, is either degraded or may serve additional regulatory roles. Deviations from this canonical pathway highlight the complexity and flexibility of miRNA biogenesis, including non-canonical pathways that bypass some steps. Importantly, miRNA expression is tissue-specific or stage-specific, emphasizing their roles in cell lineage determination and tissue-specific functions [53].

MiRNAs have diverse cellular functions, and are primarily recognized for their role in silencing and fine-tuning mRNA transcript expression. Recent advancements have unveiled a strong connection between miRNAs and the development of atherosclerosis, emphasizing their significant involvement in the disease’s pathogenesis [10]. Additionally, miRNAs have been implicated in the regulation of inflammatory responses and immune modulation, which are essential in the pathophysiology of asthma. These findings highlight the dual role of miRNAs in both cardiovascular and respiratory diseases, outlining their potential as therapeutic targets and biomarkers for complex conditions [20,47].

Epigenetic mechanisms, including DNA methylation, histone modifications and transcription factor regulation, are pivotal factors in modulating miRNA expression in both asthma and atherosclerosis [38,53,59,60]. Aberrant DNA methylation often leads to miRNA dysregulation, while the methylation status of specific miRNA genes offers prospects as a biomarker for diagnosis, but also for disease progression prediction in both conditions. Similarly, alterations in histone architecture can disrupt miRNA expression, contributing to widespread epigenetic abnormalities [35,49]. Further research into the interplay between miRNAs and epigenetic regulation is expected to drive the discovery of novel biomarkers and therapeutic targets.

Advancements in assessing the efficacy and reproducibility of miRNA biomarkers across different disease classifications and stages will enhance their clinical utility. Restoring epigenetic control of miRNA expression represents a promising therapeutic approach, utilizing drugs that inhibit DNA methylation and histone modifications. Computational gene regulation analysis offers a robust platform to investigate the intricate “epi–miR–epi” interactions involved in asthma and atherosclerosis. Integrating experimental data, clinical resources and computational tools will expand our understanding of these regulatory networks, facilitating the development of innovative diagnostic and therapeutic strategies [61,62,63,64].

To establish miRNA-based therapies as a standard treatment, further optimization of delivery methods—such as nanoparticle- and liposome-mediated systems—is essential. Ongoing and future clinical trials will be critical in validating these approaches, potentially leading to miRNA-targeted therapies in asthma and atherosclerosis management [60].

## 3. Key MiRNAs Associated with Atherosclerosis 

### 3.1. MiRNAs Involved in the Regulation of Endothelial Cell Function

Recent findings have proposed miRNAs as a novel class of inter- and intracellular regulators with significant impacts on endothelial cells (ECs) and their function. They have been linked to the pathogenesis of atherosclerosis by influencing the molecular and cellular behavior of ECs. As the initial participants in atherosclerosis, ECs undergo a series of changes under biochemical stimuli, such as increased expression of adhesion molecules like intracellular adhesion molecule (ICAM)-1, vascular adhesion molecule (VCAM)-1 and E-selectin [5,17]. These molecules facilitate leukocyte recruitment and migration to vascular margins, marking the early stages of atherosclerotic plaque formation [65]. Endothelial cell function may be altered by various stimuli, including hypoxia, inflammatory cytokines, reactive oxygen species and mechanical or metabolic factors, such as hypertension, injury, hyperglycemia or aging [53].

MiR-126, the most abundant microRNA in endothelial cells, has an important role in angiogenesis, inflammation and oxidative stress regulation, particularly in young cells. It modulates key processes by inhibiting the PI3K/Akt pathway to suppress ischemic angiogenesis and tumor growth, while activating the SIRT1/Nrf2 pathway to reduce oxidative stress and inflammation. Several miRNAs, including miR-126, miR-31 and miR-17-3p, play a significant role in modulating inflammation by regulating the expression of adhesion molecules such as ICAM-1, E-selectin and VCAM-1 [66,67,68]. MiR-126 decreases VCAM-1 expression, reducing leukocyte–EC interactions during inflammation. Reduced miR-126 levels are linked to increased VEGFA expression, which promotes vessel formation and EC migration [69]. Specific miRNAs such as miR-126 promote developmental angiogenesis by targeting Spred-1, while others, like miR-92a, inhibit angiogenesis and cell migration [70,71,72,73]. Additionally, miR-126 and other microRNAs, such as miR-21 and miR-100, are regulated by Nrf2, a key mediator of cellular responses to oxidative and inflammatory stress, linking miR-126 to the broader regulation of endothelial function and senescence pathophysiology [74]. Circulating miR-126-3p levels increase with age in healthy individuals, but not in diabetic patients, potentially contributing to higher cardiovascular risk in diabetes [75]. Elevated levels of miR-126-3p acted as a prognostic marker for cardiovascular events in a long-term study of the general population, suggesting its potential as a key indicator of endothelial cell protection to reduce the effects of vascular damage [76]. In a cohort of geriatric hospitalized patients with cardiovascular multimorbidity, reduced levels of circulating miR-17 and miR-126-3p were shown to be possible markers that may help to identify individuals at increased risk of short- and medium-term mortality [77].

MiR-21 and miR-100 are also highly expressed in EC and regulated by Nrf2. Both miR-21 and miR-100 are upregulated in senescent cells, but their levels decrease in EC exposed to pro-atherogenic stimuli, likely due to reduced Nrf2 expression [74]. MiR-21 levels may serve as a biomarker for assessing endothelial senescence. Both miRs activate the *VEGFA/MYC* pathway, influencing angiogenesis, EC proliferation and metabolic activity, but they have opposing effects—miR-21 upregulates and miR-100 downregulates the pathway [74]. Overexpression of miR-126 protects against ischemia/reperfusion injury by activating the SIRT1/Nrf2 pathway and reducing oxidative stress and inflammation, while also decreasing VCAM-1 expression to limit leukocyte adhesion [78]. MiR-100 also plays an atheroprotective role by inhibiting EC proliferation and vascular smooth muscle cell migration, with studies showing that overexpressing miR-100-5p reduces atherogenesis [79]. MiR-21 and miR-100, which show increased expression with age, may influence EC senescence by regulating angiogenesis, EC proliferation, vSMC migration, mitochondrial function and reactive oxygen species (ROS) production [74,80].

MiR-34a is overexpressed in senescent EC, and plays a key role in regulating senescence by influencing various pathways. Initially recognized as a tumor suppressor, miR-34a upregulates p53 by downregulating SIRT1, which is involved in protective mechanisms like nitric oxide (NO) production and inflammation control. Overexpression of miR-34a suppresses *SIRT1*, endothelial nitric oxide synthase (eNOS) and catalase, potentially increasing cardiovascular risk [81,82,83].

MiR-130a is downregulated in aging cells, and plays a key role in cellular proliferation, angiogenesis and protection from ischemia. Its overexpression promotes endothelial cell proliferation, migration and neovascularization by regulating proangiogenic genes [84].

MiR-146a also regulates vascular inflammation and EC activation in response to cytokines. Interestingly, its levels increase in the later stages of inflammation, and remain elevated for several days, even after pro-inflammatory cytokines are no longer present. In ECs and smooth muscle cells, miR-146a expression rises in response to shear stress, mediated by β1/β3 integrins and Nrf2, preventing excessive neointimal proliferation. Furthermore, miR-146a is overexpressed in senescent cells, potentially as a countermeasure to the heightened inflammation associated with aging [85,86,87].

While miR-146a’s role in oxidative stress response remains unclear, its overexpression in senescent endothelial cells suggests that it contributes to endothelial dysfunction by modulating vascular inflammation and proliferation [85]. This positions miR-146a as both a key player in inflammation regulation, and a potential therapeutic target for managing age-related vascular diseases and other inflammatory conditions [85,88].

The miR-200 family downregulates SIRT1, leading to increased ROS production and apoptosis, and has been linked to insulin resistance and diabetic vascular complications. In aging endothelial cells, miR-200 family members may contribute to senescence by modulating apoptosis, endothelial–mesenchymal transition and ROS production [89]. Additionally, miRs such as miR-221 and miR-222 modulate angiogenesis by targeting key factors like the c-kit receptor and eNOS, which governs NO production—a vital regulator of vascular remodeling and endothelial cell growth [90,91]. MiR-217 is highly expressed in aging ECs, and is associated with increased cardiovascular (CV) risk [92]. Its levels correlate with senescence, as it downregulates SIRT1 and influences pathways like FoxO3 and p53. Overexpression of miR-217 in senescent cells is linked to cell cycle regulation, differentiation and senescence induction [92,93,94]. It also affects NO production, extracellular matrix structure and function. In vivo, miR-217 overexpression in mice is associated with common CV disorders, such as endothelial dysfunction, atherosclerosis and heart failure [92]. Plasma levels of miR-217 are correlated with CV risk factors, making it a potential biomarker for CV risk assessment [92,94].

Similarly, miR-129-1 and miR-133 were able to inhibit key processes involved in angiogenesis—such as cell proliferation, viability and migration—by targeting vascular endothelial growth factor receptor 2 (VEGFR2) and fibroblast growth factor receptor 1 (FGFR1) in human umbilical vein endothelial cells (HUVECs) in vitro [95]. Other miRNAs, including miR-146a, miR-147 and miR-155, contribute to vascular remodeling and atherosclerotic plaque development [96]. MiR-146a is involved in plaque destabilization and the regulation of inflammation in atherosclerosis. Its function is partly attributed to its ability to influence the activation of the nuclear factor (NF)-κB signaling pathway, a key regulator of inflammatory responses [97].

The miR-181 family consists of four members (miR-181a, -181b, -181c and -181d), with each member potentially targeting different genes and pathways. MiR-181b plays a key role in regulating inflammation. Its expression in ECs is reduced by inflammatory stimuli like TNF-α or LPS, while overexpression suppresses adhesion molecules, reducing leukocyte adhesion [98]. MiR-181b also regulates NF-κB (Nuclear Factor Kappa-Light-Chain-Enhancer of Activated B Cells) signaling, and could protect against endothelial-mesenchymal transition and pulmonary hypertension [99]. Although limited data exist, miR-181b is overexpressed in aging ECs, and may promote extracellular matrix degradation by inhibiting TIMP3 [100].

The let-7 family of miRNAs is one of the largest and most conserved families across species, consisting of 10 members (let-7a, let-7b, let-7c, let-7d, let-7e, let-7f, let-7g, let-7i, miR-98 and miR-202) that are regulated by the LIN28 protein. LIN28 influences cellular growth by upregulating key cell cycle regulators, such as MYC and Ras and the IGF-PI3K-mTOR signaling axis [101]. The family is highly expressed in cardiovascular cells, with members linked to myocardial ischemia, infarction, cardiac fibrosis and heart failure. Let-7g has an atheroprotective role and regulates processes like inflammation, monocyte adhesion, angiogenesis and apoptosis [102]. It also plays a part in endothelial–mesenchymal transition, contributing to cardiac fibrosis. Overall, the let-7 family is upregulated in senescent cells, where it may regulate cellular growth, metabolism and the cell cycle, influencing senescence pathophysiology [103].

**Figure 3:**
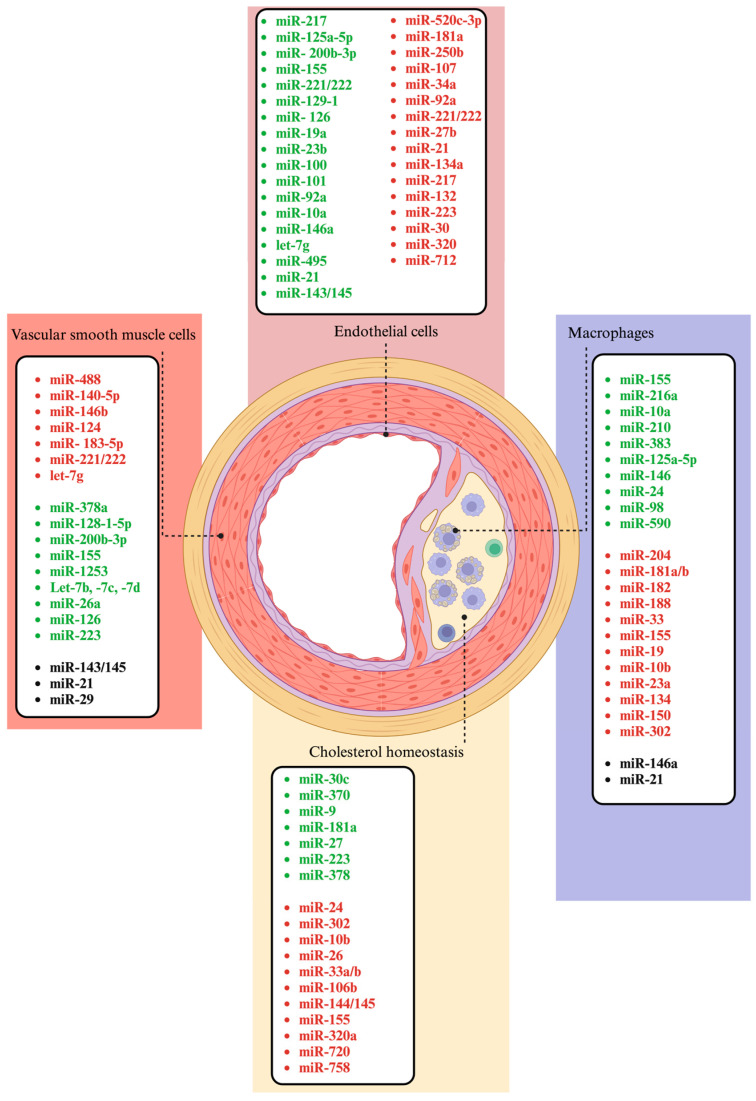
The role of microRNAs in atherosclerosis: regulation of vSMCs, ECs, macrophages and cholesterol homeostasis. vSMCs: miRNAs involved in regulating vascular contraction, relaxation and associated diseases. ECs: miRNAs critical for vascular homeostasis, angiogenesis and inflammation. Macrophages: miRNAs regulating immune response, inflammation and tissue repair. Cholesterol homeostasis: miRNAs maintaining cholesterol balance, linked to metabolic diseases and CVD. Created with BioRender.com. green: atheroprotective; red: atherogenic; black: context-dependent, showing both atherogenic and atheroprotective influences.

Shear stress—a key regulator of vascular health—also influences miRNA expression. Laminar shear stress induces atheroprotective microRNAs such as miR-10a, miR-19a and miR-143/145, while low shear stress triggers pro-atherogenic responses, exemplified by miR-21 [104]. Interestingly, miR-21 exhibits dual functions, with pro-oxidative effects under certain conditions and atheroprotective roles, such as reducing apoptosis and enhancing NO availability, under prolonged unidirectional shear stress [105]. MiRNAs also regulate the redox balance in endothelial cells by targeting key enzymes such as NOX, SOD, GPx and TrxR1. For instance, miR-25 suppression increases NOX4 expression, leading to oxidative stress, while miR-125a alleviates oxidative stress by enhancing TrxR1 activity [106]. Furthermore, miR-133 overexpression protects endothelial cells by increasing GPx activity and reducing oxidative stress-induced apoptosis [107,108,109]. MiR-148a decreases nitric oxide bioavailability and eNOS activity, which contributes to the development of early atherosclerosis. However, recent studies suggest that its effects may also include reducing inflammation; further mechanistical details on this are needed [110,111,112]. Overall, miRNAs represent a complex regulatory network influencing endothelial function, angiogenesis, redox balance and vascular remodeling, providing potential therapeutic targets for cardiovascular and atherosclerotic diseases (Figure 3).

### 3.2. MiRNAs Involved in the Regulation of Vascular Smooth Muscle Cells

VSMCs, located in the medial layer of the arterial wall, regulate vascular tone under normal conditions [113]. In atherosclerosis, vSMCs migrate from the medial layer to the intima, where they proliferate and accumulate, destabilizing plaques and increasing the risk of vascular blockages [114]. MicroRNAs have been shown to be key factors in this process, influencing vSMC behavior and atheroma progression by modulating proliferation, migration, apoptosis and inflammation [115]. These findings emphasize the dual role of miRNAs in atherosclerosis, where some act protectively by ameliorating vSMC-driven processes, while others exacerbate plaque instability and vascular damage. Vascular injury triggers rapid vascular cell proliferation and neointimal lesion formation, a process that is central to restenosis after procedures like stenting, angioplasty and arterial transplantation, as well as in subclinical atherosclerosis. A key driver is the phenotypic switch of vSMCs from a contractile to a proliferative state, which exacerbates atherosclerotic plaque development [116]. MiRNAs significantly influence vSMC behavior, plasticity and neointimal lesion formation [117]. Injury models show dynamic changes in microRNA expression, with some miRNAs downregulated (miR-125a, miR-125b, miR-133, miR-143, miR-145, miR-365) and others upregulated (miR-21, miR-146, miR-214, miR-352) [53]. MiR-192-5p targets *ATG7* to regulate autophagy, miR-214-3p downregulates *FOXO1* and miR-146b reduces the expression of Bag1 and MMP16 [87,118,119]. The MiR-143/145 cluster is highly expressed in vSMCs, and these MiRs play a significant role as regulators of vSMC differentiation, promoting a contractile phenotype and inhibiting proliferation. Their downregulation in atherosclerosis and injury models indicates their role in stabilizing plaques and controlling neointimal formation [120,121]. MiR-133 regulates vSMC phenotypic switching by repressing the anti-proliferative transcription factor Sp-1, impacting both vSMC and cardiac muscle cell functions [122,123]. The Let-7 family inhibits vSMC proliferation by targeting genes like *c-Myc*, *KRAS* and *LOX-1*, reducing intimal hyperplasia and atherosclerotic progression [103,123,124]. In addition to its role in vSMC proliferation and differentiation, miR-128 is influenced by epigenetic modifications under stress, accentuating its involvement in vascular responses to injury [125]. Recent studies suggest that miR-214 plays a role in regulating vSMC senescence, angiogenesis and proliferation. Its plasma levels are linked to these processes, indicating its contribution to vascular health [126]. Additionally, some microRNAs help to alleviate atherosclerosis by reducing apoptosis and inflammation. For instance, miR-17-5p promotes cell proliferation and wound healing, while decreasing apoptosis, by upregulating SIRT7 and inhibiting p53 activation [127]. MiR-378a, by targeting *IGF1* and toll-like receptor 8 (*TLR8)*, significantly reduces inflammation, and miR-128-1-5p inhibits inflammatory factors and apoptotic proteins via the RMRP/miR-128-1-5P/Gadd45g signaling pathway [128,129]. MiR-221 and miR-222 contribute to atherosclerosis by promoting vSMC proliferation and migration, though their precise mechanisms remain unclear [91,130].

### 3.3. MiRNAs Involved in the Regulation of Macrophages

Atherosclerosis is driven by a pro-inflammatory environment where monocytes are recruited from the circulation into areas of endothelial dysfunction and lipid accumulation [49]. Once inside the vessel wall, monocytes differentiate into macrophages, which take up lipoproteins and transform into foam cells that are specific to atherosclerosis and are major contributors to plaque formation [49]. Macrophages regulate lipoprotein uptake through scavenger receptors such as SR-A1, CD36 and LOX-1 [131]. Depending on external signals, macrophages polarize into pro-inflammatory M1 or anti-inflammatory M2 phenotypes, with M1 macrophages playing a key role in atherosclerosis progression [132].

MiRNAs are regulators of macrophage homeostasis and polarization, influencing plaque development. CD36, a key receptor facilitating oxidized LDL (ox-LDL) uptake and foam cell formation, is regulated by miRNAs such as miR-758-5p, which suppresses lipid accumulation by targeting CD36 3′UTR [133]. Additionally, miR-125a-5p and miR-146 inhibit lipid retention and inflammation by downregulating pro-inflammatory cytokines (IL-2, IL-6, TNF-α) and targeting oxysterol binding protein-like 9 (Orp-9) and TLR-4 signaling, respectively. Enhancing the expression of these miRNAs may offer atheroprotective benefits [134].

Negative feedback loops exist, where TLR stimulation induces specific miRNAs to regulate excessive inflammation. For instance, miR-147 is activated in LPS-stimulated macrophages in a TLR-4/NF-κB-dependent manner to control pro-inflammatory pathways [135]. Conversely, miR-155 is upregulated in atherosclerotic plaques, and promotes M1 macrophage polarization by increasing inflammatory cytokines (IL-6, IL-1β, TNF-α, TGF-β) and suppressing anti-inflammatory signaling molecules (BCL-6, phosphorylated STAT-3). However, studies on miR-155 inhibition in murine models have shown conflicting results regarding its effect on plaque development [136].

Another key player, miR-33, encoded within the Sterol Regulatory Element-Binding Protein 2 (*Srebf2*) gene, modulates cholesterol efflux, macrophage polarization and efferocytosis. It targets *ABCA1*, a critical cholesterol transporter for HDL-C biogenesis, thereby preventing cholesterol removal from macrophages and promoting foam cell formation. miR-33 also enhances oxidative metabolism, supporting an M1 macrophage phenotype, while anti-miR-33 treatment shifts macrophages toward the anti-inflammatory M2 phenotype, promoting plaque regression. Silencing miR-33 in atheroprone Ldlr^−/−^ mice has been shown to reduce atherosclerosis, increase macrophage apoptosis and enhance clearance [137].

Other miRNAs, including miR-128-1, miR-144 and miR-148a, also target *ABCA1*, contributing to cholesterol retention and foam cell formation. MiR-27a/b regulates genes involved in cholesterol homeostasis (*ACAT1*, *ABCA1*, *LDLR*, *CD36*) and prevents atherosclerosis by targeting lipoprotein lipase (LPL), reducing inflammation and lipid accumulation [137].

### 3.4. MiRNAs Involved in the Regulation of Inflammation

Inflammation plays a central role not only in the development and progression of atherosclerosis, but also in plaque rupture. Multiple signaling pathways drive inflammation in atherosclerosis, including ROS pathways and the mitogen-activated protein kinase (MAPK) and TLR pathways. MiRNAs regulate inflammation primarily by modulating the expression of proteins involved in these pathways [119].

A key regulator of inflammatory responses is NF-κB, which increases the production of pro-inflammatory cytokines such as TNF-α, IL-6 and IL-1β. However, its activity is modulated by NF-κB kinase inhibitors, which are direct targets of miR-223 [138]. On the other hand, miR-9 exerts anti-inflammatory effects by inhibiting NF-κB p50 expression [139]. MiR-146a-3p may suppress inflammation by targeting NF-κB during the progression of atherosclerosis, and the miR-146a-3p/NF-κB axis could serve as a promising biomarker and therapeutic target for preventing atherosclerosis and associated vascular events. A unique feature of miR-146a is its self-limiting regulatory mechanism [88,97]. For example, lipopolysaccharides (TLR4 agonists) induce miR-146a expression, which subsequently modulates downstream signaling pathways to suppress its own expression. This negative feedback loop limits the production of inflammatory molecules, tightly controlling the inflammatory response to avoid overstimulation.

ETS-1, another transcription factor regulating endothelial inflammation, is targeted by miR-221, which suppresses monocyte adhesion molecules [140]. MiR-21 promotes inflammation by upregulating VCAM-1, leading to endothelial dysfunction and leukocyte recruitment, thereby accelerating atherosclerotic plaque formation [141]. Other miRs implicated in the inflammatory response in atherosclerosis include miR-146a, miR-155, miR-126, miR-145 and miR-19 [53].

### 3.5. MiRNAs Involved in the Regulation of Cholesterol Homeostasis

Cholesterol homeostasis plays a central role in lipid accumulation within atherosclerotic plaques, and any disruption to this balance increases the risk and progression of atherosclerosis. In recent years, microRNAs have emerged as essential regulators of lipid and cholesterol homeostasis, significantly influencing the development of atheroma [142]. MicroRNA levels fluctuate in response to various stimuli. These changes can alter the activity of enzymes and regulatory factors involved in lipid metabolic pathways, thereby affecting lipid homeostasis. MiR-122 was the first identified microRNA with a role in fat metabolism, as miR-122-deficient mice exhibit impaired lipid synthesis due to enzyme deficiencies [143]. Similarly, miR-33 plays a significant role in lipid regulation by modulating cholesterol metabolism pathways and influencing beta-oxidation-related gene expression, thereby affecting fatty acid and triglyceride metabolism [144].

Other microRNAs, including miR-370, miR-130b, miR-223, miR-148a, miR-103, miR-107, miR-758 and miR-106b, also contribute to lipid metabolism regulation [145]. Disruptions to these processes can lead to excessive ox-LDL formation, which macrophages subsequently engulf, promoting lipid core enlargement in atherosclerotic plaques and further exacerbating disease progression.

For instance, miR-210-3p suppresses NF-κB activation, leading to decreased lipid accumulation and attenuated inflammatory responses [146]. MiR-34a, miR-33-5p and miR-21 contribute to inhibiting atherosclerosis progression by lowering intestinal cholesterol levels, promoting cholesterol efflux and preventing foam cell formation [144,147,148]. In addition, miR-33a/b contributes to lipid droplet accumulation by suppressing apoptosis, thereby promoting the progression of atherosclerosis [149].

MiR-7 inhibits the final steps of cholesterol synthesis by targeting genes such as DHCR24 and SC5D at the post-transcriptional level. In vivo experiments further confirmed that miR-7 reduces DHCR24 expression in the brain of mice. Additionally, cholesterol itself regulates miR-7 levels through SREBP2, which binds to its promoter. Reduced levels of miR-7 and its host gene, *hnRNPK*, were observed in models of Niemann–Pick type C1 disease and fatty liver, both characterized by excessive intracellular cholesterol [150].

Further research is needed to fully elucidate the role of microRNAs in regulating cholesterol homeostasis in atherosclerosis.

## 4. MicroRNAs as Modulators of Atherosclerosis Pathogenesis

### 4.1. MicroRNAs as Drivers of Atherosclerosis: Pro-Atherogenic Effects and Mechanisms

Pro-atherogenic miRNAs are microRNAs that drive atherogenesis, the process leading to the formation of atherosclerotic plaques in blood vessels. These miRNAs play a significant role in promoting vascular inflammation, disrupting lipid metabolism, causing endothelial dysfunction and triggering immune cell activation, all essential factors in the development and progression of atherosclerosis. The miRNAs associated with pro-atherosclerotic effects are presented in Table 1.

### 4.2. MicroRNAs as Guardians Against Atherosclerosis: Anti-Atherogenic Effects and Mechanisms

Anti-atherogenic miRNAs are microRNAs that protect against the development of atherosclerosis by counteracting the processes leading to plaque formation in blood vessels. These miRNAs play a significant role in maintaining vascular health by reducing inflammation, enhancing lipid metabolism, improving endothelial function and modulating immune responses, thereby inhibiting atherogenesis. The miRNAs associated with anti-atherosclerotic effects are listed in Table 2.

## 5. Dysregulated MicroRNAs in Asthma: Pathogenic Insights and Implications

**Figure 4:**
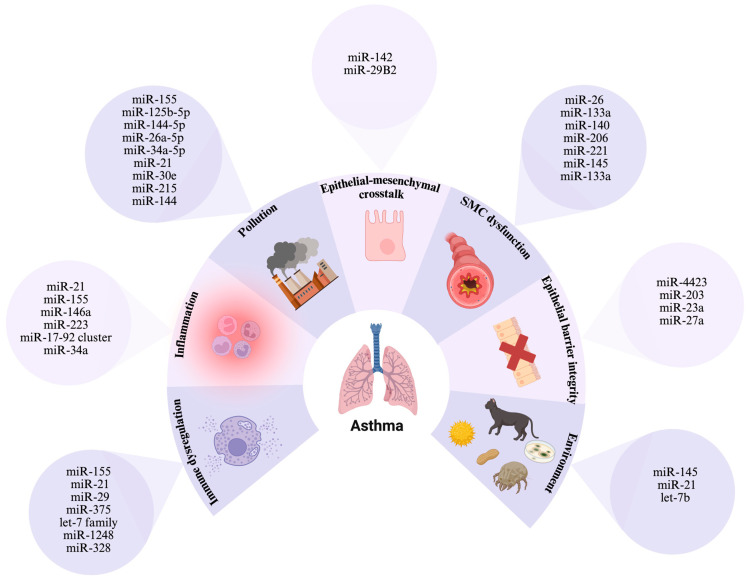
Overview of miRNAs involved in asthma-related pathways. A comprehensive list of miRNAs implicated in the regulation of asthma-related processes, including airway inflammation, bronchial hyper-responsiveness and immune modulation. MiRNAs, such as miR-155, miR-21, miR-145 and members of the let-7 family, play important roles in modulating key molecular pathways associated with the pathogenesis and progression of asthma. They are potential biomarkers for disease severity and therapeutic targets for asthma management. Created with BioRender.com.

**Figure 5:**
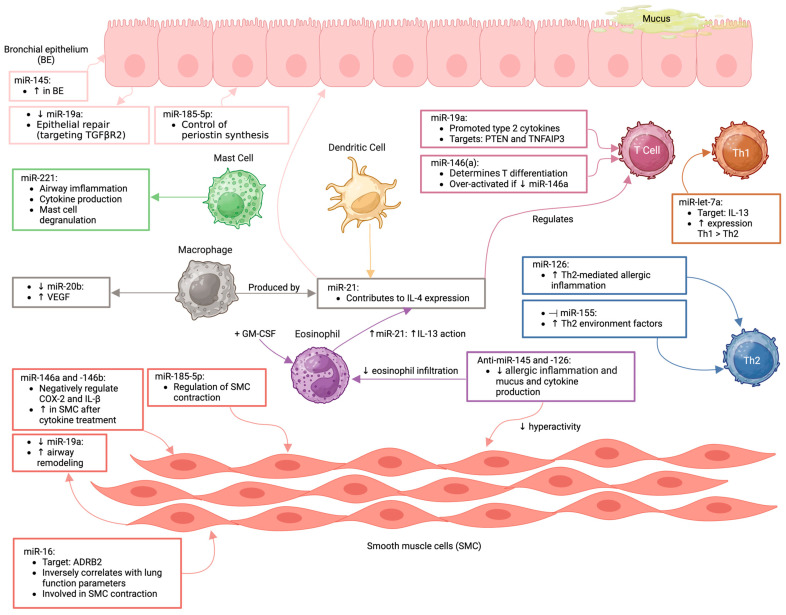
The role of microRNAs in the pathophysiological mechanisms of asthma. MiRNAs play an important role in regulating various aspects of asthma pathophysiology by modulating the functions of airway structural and immune cells. In bronchial epithelial cells, miRNAs regulate remodeling, repair processes and cytokine synthesis. Similarly, in airway smooth muscle cells, miRNAs are reported to influence contraction, hyperactivity, remodeling and cytokine production. In the immune system, miRNAs target T cells (both Th1 and Th2), particularly impacting the polarization of immune responses toward the Th2 axis, a characteristic of asthma. They also regulate eosinophil behavior, including their capacity for tissue infiltration. Furthermore, specific miRNAs control basophil degranulation and cytokine release. miRNAs also modulate the activity of macrophages and dendritic cells, particularly by influencing cytokine production. These multifaceted regulatory roles emphasize the significance of miRNAs in asthma pathophysiology. Visual elements created with BioRender.com, adapted from Gil-Martínez et al., 2023 [207].

Several miRNAs have been found to be particularly prevalent in asthma and are involved in modulating key pathways of inflammation and immune response (Figure 4 and Figure 5).

### 5.1. Inflammatory Signaling Pathways Regulated by MicroRNAs in Asthma

MiRNAs are involved in regulating the inflammatory and immune responses in asthma. They can modulate the expression of genes involved in immune cell activation, cytokine production and inflammatory signaling pathways.

MiRNAs play essential roles in macrophage polarization by influencing their pro-inflammatory or anti-inflammatory phenotypes. Specific miRNAs, including let-7f, miR-9, the miR-17~92 cluster, miR-26a/b, miR-27a/b, miR-125b and miR-155, drive macrophages towards the pro-inflammatory M1 phenotype. In contrast, miRNAs such as let-7a/b/c/d/e, miR-21, miR-34, miR-124, miR-146a/b, miR-223-3p and miR-511-3p facilitate polarization towards the anti-inflammatory M2 phenotype, reinforcing their regulatory complexity in immune responses [208]. Numerous studies have highlighted the role of miRNAs in various mechanisms underlying viral exacerbations in asthma, focusing on how miRNAs regulate host responses to specific viruses and mediate virus–host interactions. Research findings indicate that miRNAs influence antiviral immunity, control viral replication and modulate cytokine responses against respiratory viruses such as human rhinovirus (hRV), influenza virus (IV), human metapneumovirus (hMPV), human coronavirus (hCoV) and respiratory syncytial virus (RSV) [207].

### 5.2. Role in Regulating Asthma-Related Bronchoconstriction

Airway smooth muscle contraction is a characteristic of asthma and contributes to bronchoconstriction. Multiple miRNAs have been identified as key regulators of the contractile properties of airway smooth muscle cells. Notably, miR-145 and miR-133a, which are downregulated in asthmatic airways, contribute significantly to the modulation of smooth muscle cell phenotype and contractility, highlighting their potential role in asthma pathophysiology [193,209].

### 5.3. Impact on Airway Epithelial Barrier Dysfunction

The airway epithelium serves as a physical and immunological barrier, protecting against inhaled allergens, pathogens and environmental toxins. Its functionality is maintained by a complex network of processes, including epithelial cell differentiation, mucin secretion and the integrity of tight junctions, all of which are finely regulated by miRNAs. They modulate the expression of key genes essential for maintaining epithelial homeostasis, and the dysregulation of miRNAs has been shown to compromise epithelial barrier function, contributing to heightened susceptibility to external injury and chronic inflammation [36]. Among these miRNAs, miR-4423 is of particular interest due to its predominant expression in the human airway epithelium. Research demonstrates that overexpression of miR-4423 in normal human bronchial epithelial cells enhances the differentiation of ciliated cells, as evidenced by increased expression of specific ciliated cell markers. This finding demonstrates its potential role in preserving epithelial integrity and function [210].

Moreover, miR-203 has been identified as a regulator of Abelson tyrosine kinase (Abl), a gene implicated in epithelial cell behavior. By targeting *Abl*, miR-203 influences critical processes such as epithelial cell proliferation, adhesion, growth and differentiation, further highlighting the intricate role of miRNAs in epithelial biology. These insights reveal the multifaceted impact of miRNA-mediated regulation on airway epithelial health, particularly in the context of asthma, where their dysregulation can exacerbate disease pathology [211].

### 5.4. Allergic Sensitization and T Cell Polarization in Asthma Pathogenesis

Allergic sensitization, a process in which the immune system develops hypersensitivity to specific allergens, is a common and significant trigger for asthma. MiRNAs have been shown to play an important role in regulating allergic sensitization by influencing the differentiation and function of various immune cells, including T cells and dendritic cells. They can impact the immune response by modulating the balance between Th1, Th2, Th17 and Treg cell populations, all of which are key for the development and persistence of allergic inflammation [23,36,38]. Compared to healthy individuals, asthmatic individuals show a marked reduction in the levels of miRNAs such as let-7a, miR-21, miR-133a, miR-155, miR-328 and miR-1248. These changes suggest that miRNAs might contribute to the pathophysiology of asthma, potentially offering new insights for therapeutic interventions [141,212,213,214].

### 5.5. Epithelial–Mesenchymal Crosstalk

In the context of asthma, miRNAs are involved in the crosstalk between airway epithelial cells and mesenchymal cells, including fibroblasts and smooth muscle cells. This interaction is involved in the process of airway remodeling. The remodeling process includes thickening of the airway walls, smooth muscle hypertrophy and an increase in the production of extracellular matrix components, all of which can lead to airway obstruction and heightened asthma symptoms. Specifically, dysregulated expression of miRNAs can result in excessive deposition of extracellular matrix proteins and fibrosis, processes that are hallmarks of asthma exacerbations [215].

Bélanger et al. (2020) focused on the analysis of RNA samples obtained from eosinophils in individuals with atopic dermatitis, allergic rhinitis and asthma. Their study aimed to identify miRNAs that are differentially expressed across these conditions, providing insight into the shared and distinct molecular pathways involved in allergic diseases. The analysis revealed significantly differential expression of 18 miRNAs, including miR-1276, miR-29B2, miR-3175, miR-33B, miR-4308, miR-4523, miR-4673, miR-4785, miR-590, miR-638, miR-614, miR-142, miR-3064, miR-4434, miR-1304, miR-2355, miR-26A2 and miR-645. These MiRNAs were found to be differentially expressed in eosinophil samples from individuals with atopic dermatitis or asthma based on measurements of PC20 (a marker of airway hyper-responsiveness) or IgE levels, in comparison to samples obtained from healthy individuals. This study highlights how variations in miRNA expression in eosinophils may contribute to the immune dysregulation observed in asthma and allergic conditions [216]. These findings reveal the importance of miRNAs in regulating the complex interactions between immune cells, airway epithelial cells and mesenchymal cells in the context of asthma. Understanding the role of these miRNAs in allergic inflammation and airway remodeling may pave the way for novel therapeutic approaches aimed at modulating miRNA expression or function to prevent or reverse the structural changes that characterize chronic asthma. The ongoing investigation of miRNA profiles in asthma promises to expand our understanding of this complex disease and provide new avenues for targeted therapies that could improve patient outcomes.

### 5.6. The Interplay Between Oxidative Imbalance and Antioxidant Protection

MiRNAs also regulate oxidative stress and the body’s antioxidant defense mechanisms, both of which are key factors in asthma pathophysiology. Oxidative stress arises when there is an imbalance between the production of ROS and the body’s antioxidant capacity, leading to cellular damage and exacerbating airway inflammation and remodeling in asthma. Certain miRNAs, including miR-146a, miR-155, miR-128-3p and miR-21, have been shown to influence this balance by modulating the expression of antioxidant enzymes and regulating ROS-driven signaling pathways [141,217,218,219]. Through these actions, miRNAs help to control the inflammatory processes and tissue remodeling associated with asthma, serving as potential therapeutic targets for managing oxidative stress in this disease.

### 5.7. Environmental Triggers and MicroRNA-Mediated Responses

MiRNAs also mediate the effects of environmental factors, such as allergens, pollutants and respiratory viruses, on the development and progression of asthma. For example, exposure to common allergens can trigger alterations in miRNA expression profiles within the airways, leading to the disruption of immune and inflammatory responses. These changes may promote the onset of asthma or worsen its symptoms. Similarly, respiratory viruses, such as rhinovirus, have been shown to modulate miRNA expression, which can exacerbate asthma symptoms and lead to more frequent or severe exacerbations [59]. In addition to environmental influences, genetic factors also play a significant role in asthma susceptibility and severity. Specific genetic variations, such as single-nucleotide polymorphisms (SNPs) in miRNA genes or in the binding sites of their target genes, can alter miRNA binding efficiency and subsequently affect gene expression. These genetic variations can influence an individual’s susceptibility to asthma and impact the severity of the disease. Furthermore, gene–environment interactions—where genetic predisposition interacts with environmental exposures—may explain the variability in asthma susceptibility, individual responses to therapeutic interventions and the long-term outcomes of the disease. Indoor air pollution aggravates asthma in children, and induces changes in the serum level of different miRNAs such as miR-155 [220]. Thus, understanding the interplay between miRNAs, genetic factors and environmental influences is crucial for gaining a comprehensive understanding of asthma pathogenesis and for developing personalized treatment strategies [23,215].

## 6. MicroRNAs as Modulators of Asthma Pathogenesis

### 6.1. Pro-Asthmatic Pathways: Drivers of Airway Hyper-Responsiveness and Remodeling

The pro-asthmatic effects of miRNAs are largely related to their ability to modulate the function of various immune cells, promote the production of inflammatory cytokines and impact airway remodeling. The miRNAs involved in the development of asthma are summarized in Table 3.

### 6.2. Anti-Asthmatic Effects: Exploring Molecular Pathways and Functional Outcomes

Recent research has highlighted the potential of specific miRNAs in influencing asthma and its inflammatory pathways. Several miRNAs have been identified to exhibit anti-asthmatic effects by modulating immune responses, reducing inflammation, airway hyper-responsiveness and excessive mucus production. The MiRNAs associated with protection against asthma are summarized in Table 4.

## 7. Overlapping MiRNAs in Asthma and Atherosclerosis

**Figure 6:**
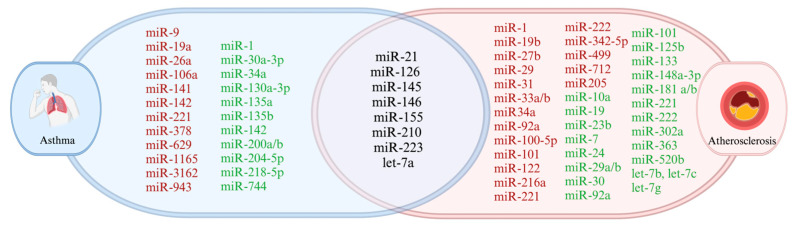
MicroRNAs at the crossroads of asthma and atherosclerosis: key regulators of shared pathophysiological mechanisms. The depicted miRNAs, such as miR-155, miR-21, miR-145 and members of the let-7 family, are involved in key processes like inflammation, immune modulation and cellular proliferation, which are common to both diseases. By modulating these pathways, miRNAs serve as potential biomarkers and therapeutic targets, offering insights into the interconnected nature of respiratory and cardiovascular diseases. Created with BioRender.com. green: predominantly protective effects; red: predominantly aggravating effects; black: common for both diseases.

MiRNAs play regulatory roles in gene expression, and have emerged as key contributors to the pathophysiology of diverse diseases, including asthma and atherosclerosis. Recent studies have highlighted overlapping miRNAs that modulate inflammatory and immune pathways that are common to both conditions, such as miR-155, miR-146a and miR-21 [23,53,245]. These miRNAs are involved in regulating macrophage polarization, T cell differentiation and cytokine production, all of which are central to the chronic inflammation observed in both diseases. Dysregulated expression of these miRNAs contributes to airway remodeling in asthma and plaque formation in atherosclerosis, drawing attention to their potential as shared biomarkers, but also as therapeutic targets [53,162,192,214]. Exploring the interaction of these miRNAs may provide novel multi-target therapeutic strategies (Figure 6).

ROS are highly reactive molecules that play essential roles in the pathogenesis of both asthma and atherosclerosis by inducing oxidative stress, inflammation and tissue remodeling. In asthma, ROS contribute to airway inflammation by increasing oxidative stress, damaging the epithelial barrier and triggering immune cell activation, leading to airway hyper-responsiveness and structural remodeling [255]. Exposure to allergens and pollutants elevates ROS levels, altering antioxidant defenses and promoting cytokine release, which amplifies inflammation and airway dysfunction [256]. Similarly, in atherosclerosis, ROS play an important role in disease progression by impairing endothelial function through NO reduction, oxidation of LDL and promoting vascular smooth muscle cell proliferation. ROS-driven activation of NADPH oxidase and inflammatory pathways further accelerates plaque formation and instability, increasing the risk of cardiovascular complications [257].

MiRNAs are regulators of ROS-mediated pathways, influencing both asthma and atherosclerosis. MiR-21 is upregulated in both diseases, promoting inflammation and fibrosis through ROS-mediated signaling, while miR-146a acts as a negative regulator of inflammation by targeting NF-κB and reducing oxidative stress [258,259,260]. MiR-155 enhances inflammatory cytokine production and immune cell activation in response to oxidative stress, whereas miR-126 plays a protective role in vascular function by maintaining endothelial integrity and mitigating ROS-induced damage [78,261].

Understanding the interplay between ROS and miRNAs in asthma and atherosclerosis provides valuable perspectives into potential therapeutic strategies. Antioxidant-based therapies, along with miRNA-targeted interventions, may offer promising alternatives for ameliorating oxidative stress and its detrimental effects in both conditions.

Pollution is a major environmental factor that exacerbates allergic asthma and cardiovascular diseases, including atherosclerosis, by influencing miRNA expression (miR-125b-5p, miR-144-5p, miR-26a-5p and miR-34a-5p), thereby driving inflammation, oxidative stress and endothelial dysfunction. MiR-26a-5p has been shown to significantly mediate air pollutant (PM_2.5_ and NO_2_)-induced effects on blood C-reactive protein (CRP) and total cholesterol levels [262].

Both miR-146a and miR-146b have been implicated in the regulation of inflammatory responses in asthma. In airway smooth muscle cells, pro-inflammatory cytokines induce the expression of these miRNAs, which, in turn, modulate the expression of inflammatory mediators such as COX-2 and IL-1 [250]. In atherosclerosis, miR-146a exhibits anti-inflammatory properties by inhibiting the NF-κB pathway and regulating cytokine expression, including IL-6, IL-8, IL-17 and TNFα. Additionally, it plays a role in vascular remodeling by modulating the proliferation and migration of vSMCs. Data suggest that it is a promising screening biomarker for atherosclerosis in the population, but it may also be a valuable therapeutic target [123].

MiR-155 is recognized for its involvement in immune regulation and inflammation. In asthma, miR-155 is upregulated, and plays a key role in allergic airway inflammation by regulating type 2 innate lymphoid cells and IL-33 signaling [263]. Research indicates that miR-155 modulates oxidative stress responses in airway epithelial cells by regulating COX-2 expression. In a mouse model of asthma, miR-155 deficiency led to reduced lung inflammation and decreased COX-2 levels, suggesting its role in promoting inflammatory pathways [218]. MiR-155 is upregulated in response to pro-inflammatory signals, promoting vascular inflammation and atherosclerosis by suppressing B-cell lymphoma 6 protein (Bcl6) in macrophages, thereby enhancing inflammatory responses. Elevated miR-155 levels contribute to endothelial cell apoptosis and inflammation, accelerating disease progression [169,264].

Elevated levels of miR-21 have been found in asthmatic patients, and are associated with higher serum IL-4 levels, suggesting that miR-21 contributes to Th2 activation and the promotion of allergic lung inflammation in asthma [265]. In atherosclerosis, miR-21 enhances proliferation and influences apoptosis in vSMCs, both of which are vital for maintaining vascular integrity and contributing to atherosclerotic plaque formation [148,154,266]. In macrophages, miR-21 is the most abundant miRNA, and its overexpression has an anti-inflammatory effect. It regulates monocyte differentiation, macrophage phenotype and macrophage-mediated inflammation, all of which influence the progression of atherosclerosis [267]. Elevated levels of miR-21 have also been observed in atherosclerotic plaques, where its deficiency in macrophages is linked to increased apoptosis and plaque instability, while overexpression in macrophages can promote a reparative, anti-inflammatory phenotype, potentially stabilizing plaques. Furthermore, circadian regulation of miR-21 expression in macrophages influences apoptosis rhythms in atherosclerotic lesions, affecting the size of the necrotic core in plaques and influencing the overall progression of atherosclerosis [268].

Elevated levels of miR-126 in the peripheral blood of children with acute asthma correlate with increased Th17 cell proportions and elevated IL-4, a representative cytokine of the Th2 response. This findings suggest that miR-126 plays a role in promoting immune dysregulation in asthma and could serve as a potential biomarker for assessing disease severity [269]. In atherosclerosis, miR-126 is essential for maintaining endothelial integrity by promoting endothelial cell proliferation and preventing apoptosis, which helps to sustain the endothelial lining of blood vessels. Disruption of this pathway, such as through the genetic deletion of Mex3a or deficiency in endothelial autophagy, leads to increased endothelial apoptosis and accelerates atherosclerosis [270,271].

MiR-145 has been identified as a significant miRNA in both asthma and atherosclerosis. In asthma, miR-145-5p is overexpressed, and plays a key role in airway remodeling by stimulating airway smooth muscle cell proliferation and migration. This occurs through the suppression of Krüppel-like factor 4 (KLF4) [230]. Additionally, in CD4+ T cells of asthma patients, miR-145-5p negatively regulates Runt-related transcription factor 3 (RUNX3), which is essential for T-helper cell differentiation. Its increased expression leads to higher IL-4 (Th2 cytokine) levels and reduced IFN-γ (Th1 cytokine) levels, shifting the immune response toward a Th2-dominant profile characteristic of allergic asthma [214]. In atherosclerosis, miR-145 plays an important role in maintaining the contractile phenotype of vSMCs [272]. On the other hand, miR-145 also targets the *ABCA1*, a regulator of cholesterol efflux, thereby influencing lipid accumulation and atherosclerotic progression [272].

MiR-221 has been implicated in asthma pathogenesis through various cellular processes. In airway smooth muscle cells, miR-221 is upregulated during disease exacerbation, where it promotes cell hyperproliferation and migration, but also reduces contractile gene markers, thereby contributing to airway remodeling [273]. Furthermore, studies have demonstrated that miR-221 is also upregulated in the airways of asthmatic mice, and is associated with increased expression of pro-inflammatory cytokines and growth factors, such as IL-6 and TGF-β, which are known to drive structural changes in the airways. In murine asthma models, inhibiting miR-221 has been shown to reduce airway hyper-responsiveness, mucus metaplasia, airway inflammation and remodeling. These findings collectively suggest that miR-221 plays a critical role in airway remodeling, potentially through its modulation of the PI3K/AKT signaling pathway [274]. In human bronchial epithelial cells, miR-221 expression is significantly higher in asthma patients, which may contribute to airway epithelial injury [275]. Additionally, miR-221-5p suppresses the Th17/Treg ratio by targeting RORγt, indicating its involvement in modulating the immune response in asthma [276]. In atherosclerosis, miR-221 promotes a phenotypic switch of vSMCs from contractile to synthetic, enhancing proliferation and motility, which are linked to neointima formation and vascular remodeling [91]. In ECs, chronic inflammation in advanced atherosclerosis downregulates miR-221, potentially activating neoangiogenesis and suggesting its role in maintaining endothelial function [91]. Loss of miR-221 in atherosclerotic plaque rupture increases p27Kip1 expression, indicating that miR-221 may affect plaque stability by regulating cell cycle progression [277].

MiR-223 is a key regulator of immune responses and airway inflammation in asthma. Elevated levels of miR-223 have been found in the peripheral blood of children with acute asthma, showing a strong correlation with increased Th17 cells and elevated IL-4 levels. These findings suggest that it may disrupt immune homeostasis, promoting a Th2/Th17-driven inflammatory response that contributes to asthma pathogenesis [278]. Inhibition of miR-223 in mouse models has been shown to reduce Th2-driven airway inflammation, indicating its role in allergic inflammation [278]. MiR-223 is also implicated in regulating pulmonary inflammation, making it a potential therapeutic target for asthma and other inflammatory diseases [279]. In atherosclerosis, miR-223 regulates lipid metabolism, inflammatory responses and macrophage activity. It protects against atherosclerosis by influencing cholesterol efflux and inflammation pathways in macrophages [132]. Additionally, miR-223-3p inhibits atherosclerosis progression by downregulating the MEK1/ERK1/2 signaling pathway in macrophages, reducing inflammation [280]. Elevated levels of miR-223-3p have also been associated with improved survival rates in atherosclerotic patients, indicating its potential as a prognostic biomarker [280].

The regulation of TGF-b signaling and SIRT-1 expression by let-7g leads to beneficial effects on ECs, and the maintenance of a sufficient let-7g level may reduce the risk of cardiovascular diseases [203].

## 8. Agomir and Antagomir Therapy: From Bench to Bedside—Unlocking the Potential of miRNA Modulation for Disease Targeting

Agomirs and antagomirs represent a transformative class of synthetic oligonucleotides engineered to modulate miRNA activity with precision. Agomirs are synthetic microRNA mimics that undergo chemical modifications to enhance the function of endogenous miRNAs. They work by increasing the activity of a specific miRNA, which results in the downregulation of its target mRNAs. Conversely, antagomirs are chemically modified antisense oligonucleotides designed to inhibit specific miRNAs, thereby blocking their function and resulting in the upregulation of their target mRNAs [281,282].

Ago/antagomir therapy holds significant potential for the treatment of diseases like asthma and atherosclerosis by modulating the expression of key miRNAs involved in inflammation, immune responses and tissue remodeling. While still in the early stages, the targeted utilization or inhibition of specific miRNAs could offer novel therapeutic avenues for managing these chronic diseases. Advancements in synthetic oligonucleotides have made therapeutic strategies utilizing antagomirs or agomirs increasingly viable.

Despite preclinical promise, key barriers persist. The transient efficacy of current miRNA modulators (e.g., 18–46 days in murine cardiac tissue and up to 28 days in pigs) necessitates innovations in sustained delivery systems, such as lipid nanoparticles or exosome-based carriers. Off-target effects remain a concern, as miRNAs often regulate multiple mRNAs; CRISPR/Cas9-based miRNA editing may offer superior specificity. Prioritizing tissue-specific targeting, for example, with lung-tropic nanoparticles for asthma or macrophage-directed therapies for atherosclerosis, will be essential to minimizing systemic toxicity [283,284].

Antagomir-21 could inhibit alveolar M2 macrophage polarization, thereby reducing Th2-driven eosinophilic airway inflammation, attenuating airway hyper-responsiveness and mitigating airway remodeling [284]. In ovalbumin-induced asthmatic mice, miR-3162-3p antagonism alleviated airway hyper-responsiveness, inflammation and Th1/Th2 cytokine imbalance, while restoring β-catenin expression. Notably, its anti-inflammatory effects were comparable to those of glucocorticoid treatment, highlighting its potential as a therapeutic target in asthma [241].

Chen et al. (2017) observed elevated expression of miR-155 in mice sensitized and challenged with ovalbumin compared to control mice. Their study further demonstrated that delivering small interfering RNA (siRNA) targeting miR-155 via a lentiviral vector effectively reduced airway inflammation and decreased levels of Th2 cytokines, including IL-4, IL-5 and IL-13, in the bronchoalveolar lavage fluid. These findings suggest that lentiviral vector-mediated delivery of siRNA targeting miR-155 holds potential as a novel therapeutic approach for treating allergic asthma [232].

Blocking miR-145-5p has been shown to restore RUNX3 levels and rebalance Th1/Th2 cytokine production [214]. Given its involvement in vSMC phenotypic modulation and lipid metabolism, miR-145 represents a promising therapeutic target. Studies have explored the use of miR-145 micelles to modulate vascular cell dynamics and ameliorate atherosclerosis, demonstrating potential in both early and advanced stages of the disease [272].

In a 2022 study, Liu et al. examined the role of miR-135b in regulating CXCL12 expression and airway inflammation in asthmatic children and mouse models. They confirmed an inverse relationship between miR-135b levels and CXCL12 expression in 68 pediatric asthma patients, linking elevated CXCL12 to bronchial hyper-reactivity and Th17-mediated inflammation. In OVA-induced asthmatic mice, treatment with miR-135b mimics reduced inflammatory cell infiltration, CXCL12 production, IL-17 levels and Th17 cell activity. These findings suggest that targeting miR-135b could offer a novel approach for managing airway inflammation in asthma [248].

MiR-126 inhibits the progression of coronary atherosclerosis in mice by binding to S1PR2, and so it offers potential for future innovative treatment options for CAD [285]. Also, it has been observed that 17β-estradiol (E2) regulates miR-126 expression, improving endothelial function and slowing the progression of atherosclerosis, possibly being mediated by Ets-1/miR-126 [286]. Therapeutic delivery of miR-126 using nanoparticle micelles has been shown to reduce atherosclerotic plaque growth by promoting the contractile phenotype of vSMCs [271].

Lipopolysaccharide stimulation increases the expression of miR-146a-5p in telocytes (TCs), a novel type of interstitial cells, leading to the suppression of the CREB1/DUOX2 pathway and a reduction in oxidative stress in cultured TCs [287]. MiR-146a balances immune responses by downregulating the NF-κB signaling pathway, including IRAK1 and TNF receptor-associated factor 6 (TRAF6), reducing airway inflammation and hyper-responsiveness [86]. Targeting miR-146a could serve as a novel therapeutic strategy for allergic asthma [250].

pH low-insertion peptide (pHLIP) could offer a promising approach for treating advanced atherosclerosis by selectively inhibiting miR-33 in macrophages, avoiding harmful effects in other metabolic tissues. This strategy has been shown to result in increased expression of fibrotic genes, such as *Col2a1*, *Col3a1*, *Col1a2*, *Fn1* and tissue inhibitor of metalloproteinase 3 (*Timp3*), while simultaneously downregulating *Mmp12*. These findings suggest that pHLIP-based delivery systems could open up new therapeutic opportunities for atherosclerosis-related cardiovascular diseases by targeting protective miRNAs directly to macrophages.

Antagomir-155 nanotherapy can reduce inflammation in atherosclerotic lesions and alleviate atherosclerosis by downregulating miR-155 and its target gene, *Bcl6*. Additionally, this treatment results in a corresponding decrease in RELA expression, as well as the downregulation of its downstream target genes, *Ccl2* and *ICAM-1* [288].

In vivo studies show that miR-127-3p agomir significantly accelerates atherosclerosis progression and macrophage proliferation, while reducing *SCD1* expression and the levels of UFAs in aortic plaques of LDLR-/- mice. In contrast, miR-127-3p antagomir attenuates atherosclerosis, reduces macrophage proliferation in LDLR-/- mice and enhances the stability of carotid plaques in mice with vulnerable plaques [289].

Systemic treatment with antagomir-142-3p reduced endothelial apoptosis and slowed the development of atherosclerosis in the aorta of ApoE-/- mice by upregulating Rictor expression and activating the Akt/eNOS signaling pathway [290].

Modulating miRNAs through agomirs or antagomirs could have systemic effects, as miRNAs regulate multiple genes and pathways across different organ systems. Endothelial-derived miRNAs have been shown to also modulate the activity of vascular smooth muscle cells, further emphasizing their role in intercellular communication and vascular homeostasis [24]. Given their role as intercellular signaling molecules, extracellular miRNAs hold potential not only as biomarkers, but also as therapeutic targets. While the dosage of a treatment is influenced by the route of administration, certain anatomical sites may benefit from a more targeted therapeutic approach through local or regional delivery. For instance, intravenous administration ensures systemic distribution, whereas respiratory, intraperitoneal, intracranial or other localized routes can provide regional effects, thereby enhancing therapeutic efficacy while minimizing systemic adverse effects. Some key systemic effects could include the immune system modulation, as exemplified by miR-155, miR-21 and miR-146a, which influence immune cell differentiation and cytokine production. Their modulation could reshape systemic inflammatory responses, potentially influencing the pathogenesis of both autoimmune and allergic diseases [291]. Additionally, the role of miRNAs in maintaining cardiovascular homeostasis has been extensively investigated. The systemic administration of miR modulators may affect blood pressure regulation, vascular remodeling, atherosclerotic plaque stability and thrombotic risk, influencing overall cardiovascular health [188,292]. The modulation of lipid metabolism and insulin sensitivity by miRNAs may influence metabolic disorders such as diabetes and obesity, conditions that share inflammatory pathways with asthma and atherosclerosis. Furthermore, considering the broad regulatory functions of miRNAs, systemic administration of miR-based therapies may lead to unintended gene regulation in non-target tissues, necessitating precise delivery strategies to avoid adverse effects [291].

Addressing off-target effects and potential immunogenicity remains a priority. Optimizing delivery methods to target specific tissues without systemic side effects is critical. Clinical trials are needed to establish the safety and efficacy of antagomir therapies in human populations. Clinical trials specifically targeting miRNAs in asthma and atherosclerosis are currently limited, but the advancement of miRNA-based therapeutics in other diseases emphasizes their potential applicability to these conditions. Understanding the role of miRNAs in inflammatory pathways could lead to novel treatments for both asthma and atherosclerosis. Several miRNA-based drugs have progressed to Phase I and Phase II clinical trials targeting various diseases [293,294]. Table 5 summarizes the ongoing and recent clinical trials investigating miRNA targets relevant to asthma and atherosclerosis.

## 9. Conclusion and Future Perspectives: Bridging Asthma and Atherosclerosis Through MiRNA Signaling

Emerging evidence positions miRNAs as central molecular regulators connecting asthma and atherosclerosis through shared inflammatory and remodeling pathways. This connection emphasizes the potential for developing integrated therapeutic approaches targeting these microRNAs to simultaneously address both diseases. Further investigation into their precise roles could pave the way for novel treatment strategies that target the underlying molecular mechanisms common to both conditions.

Key microRNAs such as miR-21, miR-126, miR-145, miR-146, miR-155, miR-221 and miR-223 orchestrate overlapping processes: Th1/Th2/Th17 polarization, vSMC phenotypic switching, oxidative stress and extracellular matrix dysregulation. Targeting these miRNAs presents a promising therapeutic approach for treating both conditions simultaneously. However, miRNA-based treatments, such as agomir and antagomir therapies, are still in the early stages, and face challenges related to efficient delivery and off-target effects.

Emerging advancements in miRNA research offer transformative opportunities for the clinical translation of asthma and atherosclerosis therapies. Profiling circulating miRNAs, such as miR-126, miR-155 and miR-223, which could serve as dual-target biomarkers, may enable early detection of comorbid asthma–atherosclerosis phenotypes and guide personalized therapeutic strategies. Combinatorial delivery systems, such as co-administering antagomirs (e.g., miR-21/miR-155) with statins or biologics (e.g., an-ti-IL-4Rα), hold promise for synergistically suppressing inflammation while reducing drug resistance.

Long-term studies are essential to assess the safety and efficacy of miRNA therapies in clinical settings. Additionally, further research into how asthma and atherosclerosis influence each other will help to refine treatment approaches for patients with both conditions.

In conclusion, miRNAs represent a promising avenue for treating asthma and atherosclerosis by targeting shared molecular mechanisms. Continued research will be essential in translating these insights into effective therapies. MiRNA-based therapeutics hold transformative potential for bridging asthma and atherosclerosis management. By targeting conserved inflammatory pathways, these molecules could redefine precision medicine for chronic inflammatory diseases. However, their success hinges on interdisciplinary collaboration, spanning bioengineering, immunology and clinical trial design, to address existing translational challenges and accelerate the path to their clinical application.

## Figures and Tables

**Table 1:** MiRNAs associated with pro-atherosclerotic effects.

MiRNA	Effect	Target/Pathway	References
miR-1	Induction of vSMC differentiation, promotion of inflammation	*KLF4*, NF-κB	[151]
miR-19b	Promotion of macrophage foam cell formation	*ABCA1*, PPARγ	[152,153]
miR-21	Promotion of EC inflammatory phenotype	NF-κB	[154]
	Stimulation of vSMC proliferation	*PTEN*, *SPRY1*, *RASA1*	[104]
miR-27b	Plaque progression and development	*Pparg*, *Angptl3*, *Gpam*	[155,156]
miR-29	Mediation of vSMC calcification	ADAMTS-7	[157]
miR-31	Regulation of macrophage cell proliferation and apoptosis	NOX4	[67]
miR-33a/b	Regulation of cholesterol and fatty acid homeostasis	*ABCA1*, *ABCG1*	[142,158]
miR34a	Promotion of EC senescence	SIRT1	[159,160]
miR-92a	Induction of endothelial dysfunction and inflammation, inhibits angiogenesis	KLF2, KLF4, SOCS5	[161,162,163]
miR-100-5p	Inhibition of cell progression and inflammatory response in eosinophils	FZD5/Wnt/β-catenin pathway	[79]
miR-101	Promotion of foam cell formation	*ABCA1*	[164]
miR-122	Regulation of cholesterol and fatty acid homeostasis	HMGCR, SREBP2, fatty acid synthase	[165]
miR-145	Regulation of vSMC phenotypic modulation	KLF4/KLF5, ELK1, *ABCA1*/SCARB1	[121]
miR-155	Promotion of inflammation	*Bcl6*	[166,167,168,169,170]
	Enhancement of inflammation, impairment of cholesterol efflux	SOCS1	
	Suppression of anti-inflammatory signals	BCL-6, pSTAT3	
miR-210	Induction of EC apoptosis	PDK1	[171]
miR-216a	Promotion of senescence of EC	SMAD3/*IκBα*	[172]
miR-221	Induction of EC dysfunction	AMPK	[173,174]
miR-222	Promotion of vSMC proliferation and migration	eNOS	[175]
miR-223	Promotion of EC apoptosis Thrombus formation	HMG-CoA synthase 1, methyl sterol, monooxygenase 1	[132]
miR-342-5p	Enhancement of the inflammatory stimulation of macrophages	AKT1	[176]
miR-499	Promotion of vSMC proliferation and migration	SOX6	[177]
miR-712-miR205	Stimulation of pro-inflammatory EC responses	TIMP3	[178]

**Table 2:** MiRNAs associated with anti-atherosclerotic effects.

MiRNA	Effect	Target/Pathway	References
miR-10a	Anti-inflammatory effect	MAP3K7, βTRC	[179]
	Antiapoptotic effect	*Bim1*	
miR-19	Inhibition of shear stress-induced cellular proliferation	cyclin D1	[180]
miR-21	Reduces plaque necrosis and inflammation	MAP2K3	[181]
	Enhances EC survival by decreasing apoptosis and activating the NO Pathway	EC	[154]
miR-23b	Inhibits EC proliferation	E2F1, Rb	[182]
miR-7	Inhibits the final steps of cholesterol synthesis	DHCR24, SC5D, SREBP2	[150]
miR-24	Inhibits the proliferation and migration of ECs	importin-α3, MMP14	[183]
miR-29a/b	Inhibition of calcification of vSMCs	ADAMTS-7	[157]
	Regulates cell migration	MMP-2/MMP-9	[184]
miR-30	Regulation of endothelial cell autophagy	ATG6	[185]
miR-92a	Blocks angiogenesis and reduces migration of endothelial cells	Integrin-alpha5	[186]
miR-101	Regulates Vascular Smooth Muscle Cell Migration	DOCK4	[187]
miR-125b	Inhibition of calcification of vSMCs	*Ets-1*, SP-7	[188,189]
miR-126	Inhibits apoptosis of EC	SIRT1, SOD2	[190]
	Reduction of lipid accumulation	*Mac*	[191]
	Reduction of inflammatory response	*MAP3K10*	[192]
miR-133	Reduction of oxidative stress-induced apoptosis	caspase-9/caspase-3	[11]
	Suppression of EC proliferation rate, viability and migration activity	VEGFR2 and FGFR1	[95]
miR-143/145 cluster	Inhibition of vSMC proliferation	KLF4, PDGFR-β, TPM4	[120,193]
	Promotion of the contractile phenotype of vSMCs	ELK1	
	Reduction of inflammatory response	ADAM17	
miR-145	Regulates vSMC fate and plasticity	KLF4	[193]
miR-146a	Delays endothelial cell senescence Delays both inflammatory response and ox-LDL accumulation	NOX4 TRAF6/IRAK1 TLR4	[85,88,194]
miR-148a-3p	Inhibition of ox-LDL-induced cell apoptosis and inflammation	CNTN4	[111]
miR-155	Reduction of inflammation	MAP3K10	[195,196]
	Enhancement of cholesterol efflux from macrophages	SOCS1	
	Internalization and endocytosis of oxidized LDL in macrophages	CD36, *Vav3*	
miR-181a/b	Modulates endothelial integrity and reduces inflammation	TAB2, NEMO, MAP3K3, importin-α3, TIMP3/ELN	[197,198]
miR-210	Attenuation of lipid accumulation and inflammation	IGF2	[146]
miR-221	Inhibition of endothelial progenitor cell proliferation	PAK1	[174]
miR-222	Enhances anti-inflammatory response	SOCS3/JAK2/STAT3	[199]
miR-223	Regulation of cholesterol metabolism and inflammation	*ABCA1*, IKKα, NLRP3	[132]
miR-302a	Modulation of cholesterol efflux	*ABCA1*	[200]
miR-363	Reduction of EC inflammatory responses	NOX4	[201]
miR-520b	Inhibition of endothelial activation	NF-κB/p65/ICAM1/VCAM1	[202]
let-7g	Inhibition of foam cell formation and adipocyte differentiation	HMGA2/CEBPβ	[203]
let-7a	Inhibition of ox-LDL-induced endothelial cell apoptosis, NO deficiency, ROS overproduction, LOX-1 upregulation and eNOS downregulation	LOX-1	[204]
let-7b, let-7c, let-7c	Inhibition of EC apoptosis and dysfunction, vSMC migration/proliferation Attenuation of pro-inflammatory cytokine release from human plaques Reduction of plaque size in vivo	LOX-1, HAS-2	[204,205,206]

**Table 3:** MiRNAs associated with pro-asthmatic effects.

MiRNA	Effect	Target/Pathway	References
miR-9	Promotion of inflammatory responses	Protein phosphatase 2A regulatory subunit B, SLC26A2	[221]
miR-19a	Inhibition of proliferation and migration of airway smooth muscle cells (aSMCs)	PTEN	[222]
miR-21	Enhancement of Th2 polarization by suppressing Th1-polarizing cytokines and allergic airway inflammation	PDCD4 PTEN	[223,224]
miR-26a	Promotion of aSMC hypertrophy	GSK-3β	[225]
miR-106a	Regulation of immune pathways	IL-10	[164]
miR-126	Modulation of gene translation to bridge TLR4/ MyD88 signaling and Th2 response in a BALB/c mouse model of HDM-induced allergic asthma	OBF.1→ ↓ GATA3	[226]
miR-141	Stimulation of IL-13-induced goblet cell metaplasia, promotes airway remodeling and pathological airway mucus production	CLCA1	[227]
miR-142	Enhances FcεRI-mediated degranulation and rescues the reduction of degranulation Modulation of M2 polarization and profibrotic activities	LPP SOCS1	[228]
miR-145-5p	Enhancement of eosinophilic inflammation, mucus hypersecretion, AHR and Th2 cytokine production Stimulation of epithelial barrier dysfunction and suppression of epithelial repair	KLF 4/5 RUNX3 ↓ KIF3A	[229,230]
miR-155	Enhancement of airway remodeling Modulation of Th2 responses through the transcription factor PU.1 Enhancement of IL-4, -5, -13 and -17a secretion; enhancement of mucus secretion; stimulation of T cell activation and proliferative response	↓ SOCS1, *Pu.1*, *c-Maf* ↓ COX-2 MAF bZIP transcription factor CTLA-4	[218,231,232,233,234]
miR-221	Reduces the permeability of inflammatory cells, regulates the cell cycle of mast cells and promotes their proliferation; increases IL-4 secretion and promotes the differentiation of Th cells into Th2 cells	P27KIP1; PTEN and p38/NF-κB pathway	[235,236]
miR-223	Inhibition of airway inflammation, NLRP3 levels and IL-1β release	NLRP3	[237]
miR-378	Promotion of aSMC proliferation and apoptosis resistance	ErbB, RAS, MAPK and calcium signaling pathway	[238]
miR-629	Enhancement of IL-1β and IL-8 protein, which show a positive correlation with increased neutrophil presence in sputum	IL-8 mRNA	[239]
miR-1165	Inhibition of bronchial hyper-responsiveness, airway inflammation and differentiation of T cells toward Th2	IL-13, PPM1A	[240]
miR-3162	Enhancement of bronchial hyper-responsiveness and airway inflammation; regulation of Th1/Th2 balance	β-catenin	[241]
miR-943	Increases the number of macrophages, eosinophils, lymphocytes and neutrophils; enhances the expression of collagen, β-catenin and c-Myc in lung tissue; promotes subepithelial fibrosis and aSMC proliferation	SFRP4	[242]

**Table 4:** MiRNAs associated with anti-asthmatic effects.

MiRNA	Effect	Target/Pathway	References
Let-7a	Inhibits proliferation and promotes apoptosis of human aSMCs	STAT3	[213]
miR-1	Inhibits secretion of IL-4, -5, -8 and TNF-α; regulates Th1/Th2 balance	-	[243]
miR-30a-3p	Inhibition of the secretion of specific IgE, eotaxin-1, IL-5 and IL-4	CCR3	[244]
miR-34a	Enhancement of the polarization of Th2 cells	Wnt signaling pathway	[245]
miR-130a-3p	Modulation of M2 polarization and profibrotic activities	Proliferator-activated receptor γ	[246]
miR-135a	Inhibition of bronchial hyper-responsiveness and lung pathological changes; reduction of the secretion of TNF-α, IL-6, IL-5 and Eotaxin	STAT family	[247]
miR-135b	Inhibition of the immune response of Th17 cells, goblet cell proliferation and bronchial hyper-responsiveness; reduction of the number of eosinophils and lymphocytes	CXCL12	[248]
miR-142	Regulation of airway inflammation	PTEN/AKT	[249]
miR-146a/b	Regulation of inflammatory responses	IRAK1, TRAF6	[250]
miR-200a/b	Inhibition of the secretion of TNF-α, IL-4, -5, -13 and -1β	ORMDL3	[251]
miR-204-5p	Inhibition of aSMC proliferation and extracellular matrix production	SIX1	[252]
miR-210	Enhancement of the polarization of Th2 cells	FOXP3	[245]
miR-218-5p	Inhibition of bronchial hyper-responsiveness, eosinophilic airway inflammation and the expression of CCL26	CTNND2	[253]
miR-744	Inhibition of bronchial epithelial cell proliferation	TGF-β1	[254]

**Table 5:** Clinical trials targeting miRNAs in asthma and/or atherosclerosis.

MiRNA	Title	Status	Identifier	Sponsor	Enrollment	Study Type	References
miR-365-3p	The Relationship of Platelet Micro-RNA Expression and Platelet Reactivity in Patients Under Clopidogrel or Ticagrelor Treatment	Completed (2017)	NCT02101437	Taipei City Hospital	175	Interventional	[295] *
panel of miRNAs	Circulating microRNAs and Adverse Cardiovascular Outcomes in Patients With Coronary Artery Disease	Recruiting	NCT03635255	National Taiwan University Hospital	460 (estimated)	Observational	*
miRNA 92a miRNA 210	Extracellular microRNA: Biomarkers of Endothelial Dysfunction in Obese Adolescents & Adults With Obstructive Sleep Apnea	Unknown	NCT03546751	University of California, San Diego	100 (estimated)	Interventional	*
panel of miRNAs	Study of microRNAs in the Evolution of Carotid Plaque. Physiopathological and Therapeutic Interest	Unknown	NCT03149406	Centre Hospitalier Universitaire, Amiens	245 (estimated)	Observational	*
miR-100, miR-125a, miR-127, miR-133a, miR-145, miR-221	Physiopathological and Therapeutic Value of microRNA in the Progression of Carotid Artery Plaques: Carotid Protocol and microRNA (CAMIA2)	Unknown	NCT02804659	Centre Hospitalier Universitaire, Amiens	120 (estimated)	Observational	*
panel of miRNAs	microRNAs in the Diagnosis of Atherosclerotic Plaque Instability	Unknown	NCT05680935	I.M. Sechenov First Moscow State Medical University	60 (estimated)	Interventional	*
panel of miRNAs	Pathogenic Mechanisms of Cancer and Cardiovascular Diseases	Completed	NCT03051191	Sakakibara Heart Institute	66	Observational	[296] *
miR-146a, miR-126	Interplay of miRNA-146a and miRNA-126 in Chronic Periodontitis Patients With Coronary Artery Disease	Completed	NCT04583085	Meenakshi Ammal Dental College and Hospital	75	Observational	[297] *
panel of miRNAs	miRNAs in Critical Limb Ischemia (miRNACLI) (miRNACLI)	Recruiting	NCT06066268	IRCCS Policlinico S. Donato	80 (estimated)	Observational	*
panel of miRNAs	The Effect of Structured Lifestyle Modification and Yoga Practice on Metabolic Processes Associated With Cardiovascular Disease (SLYM II)	Recruiting	NCT05250310	Icahn School of Medicine at Mount Sinai	120 (estimated)	Interventional	*
panel of miRNAs	RNA Sequencing in the Framingham Heart Study Third Generation Cohort Exam 2	Completed	NCT03225183	National Heart, Lung, and Blood Institute (NHLBI)	1700	Observational	*
panel of miRNAs	Extracellular RNAs in Relation to Cardiometabolic Risk	Completed	NCT03225196	National Heart, Lung, and Blood Institute (NHLBI)	4495	Observational	*
panel of miRNAs	Circulating microRNAs and Degenerative Abdominal Aorta Aneurysm (ACTA-miRNA)	Completed	NCT03974958	Assistance Publique Hopitaux De Marseille	106	Interventional	*
panel of miRNAs	Assessment of the TGF-beta Pathway and Micro-RNA in Pediatric Pulmonary Arterial Hypertension	Unknown	NCT04489251	Medical College of Wisconsin	40 (estimated)	Observational	*
miR-8059	MAP-Calcification: MicroRNAs as Potential Biomarkers for Coronary Artery Calcification	Completed	NCT01992848	University of Surrey	45	Interventional	[298] *
panel of miRNAs	Potential Diagnostic and Prognostic Value of microRNAs for the Patients of Acute Coronary Syndrome	Unknown	NCT02755207	Xinhua Hospital, Shanghai Jiao Tong University School of Medicine	100 (estimated)	Observational	*
miR-32	Coronary Artery Calcification in Type 2 Diabetes Mellitus (USCAC Study)	Recruiting	NCT04889053	University of South China	1400 (estimated)	Observational	*
miR-210	The Role of microRNA-210 in Regulating Oxidative Stress in Patients With PAD	Active, not recruiting	NCT04089943	University of West Florida	230	Interventional	*
panel of ~200 miRNAs	Role of microRNAs in T Cell-Driven Inflammation in Asthma (RITA)	Completed	NCT01484691	University of California, San Francisco	55	Interventional	*
miR-141	Study of the Inflammation and Airway Changes That Occur After Exposure to Allergen in Asthmatics (ACE)	Completed	NCT02230189	University of California, San Francisco	28	Interventional	[227] *
miR-7, miR-20a, miR-21, miR-22, miR-145 and miR-155	Circulating Micro RNAs Expression in Egyptian Bronchial Asthma and COPD Patients	Completed	NCT02719145	Tanta University	30	Observational	[299] *
panel of miRNAs	Interaction Between Benralizumab and Basophils in Eosinophilic Asthma (BASEAS)	Completed	NCT04742504	Instituto de Investigación Sanitaria de la Fundación Jiménez Díaz	20	Observational	*

* All data were collected from https://clinicaltrials.gov/ (accessed on 6 April 2025).

## Abbreviations

ABCA1	ATP-Binding Cassette Subfamily A Member 1
ABCG1	ATP-Binding Cassette Subfamily G Member 1
ADAM17	ADAM Metallopeptidase Domain 17
ADAMTS-7	A disintegrin and metalloproteinase with thrombospondin motifs 7
AKT	Protein Kinase B
AMPK	AMP-Activated Protein Kinase
Angptl3	Angiopoietin-like 3
ATG6	Autophagy-Related 6 (also known as Beclin-1)
BCL-6	B-cell lymphoma 6
Bim1	BCL2 Interacting Mediator of Cell Death
c-Maf	c-Maf Transcription Factor
CCR3	C-C Motif Chemokine Receptor 3
CD	Cluster of Differentiation
CEBPβ	CCAAT/enhancer-binding protein beta
CLCA1	Chloride Channel Accessory 1
CNTN4	Contactin 4
COX-2	Cyclooxygenase-2
CTLA-4	Cytotoxic T-Lymphocyte Antigen 4
CTNND2	Catenin Delta 2
CXCL12	C-X-C Motif Chemokine Ligand 12 (also known as SDF-1)
DHCR24	24-Dehydrocholesterol Reductase
DOCK4	Dedicator of Cytokinesis 4
E2F1	E2F1 Transcription Factor
EC	Endothelial Cells
ELK1	ETS-Like Protein 1
ELN	Elastin
eNOS	Endothelial Nitric Oxide Synthase
ErbB	Erb-B Receptor Tyrosine Kinase Family
Ets-1	Ets-1 Transcription Factor
FGFR1	Fibroblast Growth Factor Receptor 1
FOXP3	Forkhead Box P3
Gpam	Glycerol-3-phosphate acyltransferase
GSK-3β	Glycogen Synthase Kinase 3 Beta
HAS-2	Hyaluronan Synthase 2
HMG-CoA	3-Hydroxy-3-Methylglutaryl-Coenzyme A
HMGA2	High Mobility Group AT-Hook 2
HMGCR	3-Hydroxy-3-Methylglutaryl-CoA Reductase
ICAM1	Intercellular Adhesion Molecule 1
IGF2	Insulin-like Growth Factor 2
IKKα	IκB Kinase Alpha
IL	Interleukin
IRAK1	Interleukin-1 Receptor-Associated Kinase 1
IκBα	Inhibitor of kappa B Alpha
JAK2	Janus Kinase 2
KIF3A	Kinesin Family Member 3A
KLF2	Kruppel-Like Factor 2
KLF4	Krüppel-like factor 4
LOX-1	Lectin-like Oxidized LDL Receptor-1
LPP	LIM Domain-Containing Protein
MAP2K3	Mitogen-Activated Protein Kinase Kinase 3
MAP3K10	Mitogen-Activated Protein Kinase Kinase Kinase 10
MAP3K3	Mitogen-Activated Protein Kinase Kinase Kinase 3
MAP3K7	Mitogen-Activated Protein Kinase Kinase Kinase 7
MAPK	Mitogen-Activated Protein Kinase
MMP	Matrix Metalloproteinases
NEMO	NF-κB Essential Modulator
NF-κB	Nuclear Factor Kappa-Light-Chain-Enhancer of Activated B Cells
NLRP3	NOD-, LRR- and pyrin domain-containing protein 3
NOX4	NADPH oxidase 4
OBF 1	Oct binding factor 1
ORMDL3	ORM1-like 3
P27KIP1	Cyclin-Dependent Kinase Inhibitor 1B
PAK1	p21-Activated Kinase 1
PDCD4	Programmed cell death protein 4
PDGFR-β	Platelet-Derived Growth Factor Receptor Beta
PDK1	3-Phosphoinositide-Dependent Protein Kinase-1
PP2A	Protein phosphatase 2A regulatory subunit B
Pparg	Peroxisome Proliferator-Activated Receptor Gamma
PPARγ	Peroxisome Proliferator-Activated Receptor Gamma
PPM1A	Protein Phosphatase, Magnesium-Dependent 1A
pSTAT3	Phosphorylated Signal Transducer and Activator of Transcription 3
PTEN	Phosphatase and Tensin Homolog
Pu.1	Purine Rich Box-1 Transcription Factor
RAS	Rat Sarcoma
RASA1	RAS p21 Protein Activator 1
Rb	Retinoblastoma Protein
RUNX3	Runt-related transcription factor 3
SC5D	Sterol-C5-Desaturase
SCARB1	Scavenger Receptor Class B Type 1
SFRP4	Secreted Frizzled-Related Protein 4
SIRT1	Sirtuin 1
SIX1	SIX Homeobox 1
SLC26A2	Solute Carrier Family 26 Member 2
SMAD3	SMAD Family Member 3
SOCS1	Suppressor of Cytokine Signaling 1
SOCS3	Suppressor of Cytokine Signaling 3
SOCS5	Suppressor of Cytokine Signaling 5
SOD2	Superoxide Dismutase 2
SOX6	SRY-Box Transcription Factor 6
SP-7	Osterix
SPRY1	Sprouty Homolog 1
SREBP2	Sterol Regulatory Element-Binding Protein 2
STATs	Signal Transducers and Activators of Transcription
TAB2	TAK1 Binding Protein 2
TGF-β1	Transforming Growth Factor Beta 1
TIMP3	Tissue Inhibitor of Metalloproteinases 3
TLR4	Toll-Like Receptor 4
TPM4	Tropomyosin 4
TRAF6	TNF Receptor-Associated Factor 6
Vav3	Vav3 Guanine Nucleotide Exchange Factor
VCAM1	Vascular Cell Adhesion Molecule 1
VEGFR2	Vascular Endothelial Growth Factor Receptor 2
vSMC	Vascular smooth muscle cell
βTRC	Beta-Transducin Repeat-Containing
